# The unfolded protein response affects readthrough of premature termination codons

**DOI:** 10.1002/emmm.201303347

**Published:** 2014-04-04

**Authors:** Yifat S Oren, Michelle L McClure, Steven M Rowe, Eric J Sorscher, Assaf C Bester, Miriam Manor, Eitan Kerem, Joseph Rivlin, Fouad Zahdeh, Matthias Mann, Tamar Geiger, Batsheva Kerem

**Affiliations:** 1 Department of Genetics, The Hebrew UniversityJerusalem, Israel; 2 Gregory Fleming James Cystic Fibrosis Research Center, University of Alabama at BirminghamBirmingham, AL, USA; 3 Cystic Fibrosis Center, Hadassah University HospitalJerusalem, Israel; 4 The Cystic Fibrosis Center, Carmel HospitalHaifa, Israel; 5 Department of Proteomics and Signal Transduction, Max Planck Institute for BiochemistryMartinsried, Germany; 6 Department of Human Molecular Genetics and Biochemistry, Tel Aviv UniversityTel Aviv, Israel

**Keywords:** nonsense-mediated mRNA decay, premature termination codon, readthrough treatment, unfolded protein response

## Abstract

One-third of monogenic inherited diseases result from premature termination codons (PTCs). Readthrough of in-frame PTCs enables synthesis of full-length functional proteins. However, extended variability in the response to readthrough treatment is found among patients, which correlates with the level of nonsense transcripts. Here, we aimed to reveal cellular pathways affecting this inter-patient variability. We show that activation of the unfolded protein response (UPR) governs the response to readthrough treatment by regulating the levels of transcripts carrying PTCs. Quantitative proteomic analyses showed substantial differences in UPR activation between patients carrying PTCs, correlating with their response. We further found a significant inverse correlation between the UPR and nonsense-mediated mRNA decay (NMD), suggesting a feedback loop between these homeostatic pathways. We uncovered and characterized the mechanism underlying this NMD-UPR feedback loop, which augments both UPR activation and NMD attenuation. Importantly, this feedback loop enhances the response to readthrough treatment, highlighting its clinical importance. Altogether, our study demonstrates the importance of the UPR and its regulatory network for genetic diseases caused by PTCs and for cell homeostasis under normal conditions.

## Introduction

About 30% of inherited and acquired diseases are attributable to premature termination codon (PTC) through nonsense or frameshift mutations (Mendell & Dietz, 2001; Kuzmiak & Maquat, 2006). In recent years, an extensive effort has been made to develop therapeutic approaches for in-frame PTCs aimed to promote translational readthrough of the PTC and generate full-length functional proteins. One such approach is readthrough by aminoglycoside antibiotics (Burke & Mogg, 1985; Martin *et al*, 1989), synthetic aminoglycosides (Rowe *et al*, 2011), and ataluren (PTC124) (Hirawat *et al*, 2007; Welch *et al*, 2007; Kerem *et al*, 2008) that lead to translational readthrough of PTCs by the insertion of an amino acid at the stop codon (Burke & Mogg, 1985; Martin *et al*, 1989; Van de Peer *et al*, 1994; Fourmy *et al*, 1996; Recht *et al*, 1996, 1999; Fan-Minogue & Bedwell, 2007; Keeling *et al*, 2012). Hence, protein translation can continue to the normal end of the transcript and a full-length protein is generated. Many cancers are associated with PTCs. In terms of their potential as anti-cancer agents, aminoglycosides have been shown to interfere with the oncogenic process both in culture cells and in mouse models (Bidou *et al*, 2012). Readthrough studies performed in several colorectal cancer models demonstrated the restoration of mutant APC, resulting in reduced oncogenic phenotypes both *in vitro* and *in vivo* (Zilberberg *et al*, 2010; Floquet *et al*, 2011; Bordeira-Carrico *et al*, 2012). Recently, several readthrough compounds have been shown to enable repair of UV damage in xeroderma pigmentosum (XP) cells (Kuschal *et al*, 2013). These studies demonstrate the therapeutic potential of readthrough for treating cancer.

Readthrough studies using aminoglycosides or ataluren, performed in our center as well as in others, have revealed variable responses to the treatment among cystic fibrosis (CF) patients carrying PTCs (Wilschanski *et al*, 2000, 2003; Clancy *et al*, 2007; Kerem *et al*, 2008; Sermet-Gaudelus *et al*, 2010). A variable response was also observed among Duchenne muscular dystrophy (DMD) patients carrying a PTC in the dystrophin gene and in a DMD mouse model (Barton-Davis *et al*, 1999; Wagner *et al*, 2001; Dunant *et al*, 2003; Politano *et al*, 2003). Readthrough efficiency is influenced by several factors such as the PTC identity and the sequences surrounding the PTC especially the fourth nucleotide immediately after the stop codon (Linde & Kerem, 2008). Another important factor is the level of transcripts available for readthrough. We have previously found an extended variability among CF patients in the nonsense transcript level, which was correlated with their response. A response was found only in patients with relatively high levels of CFTR transcripts (Linde *et al*, 2007b; Kerem *et al*, 2008). Since the transcripts serve as templates to the readthrough process, their level is a limiting factor of the response. Different cellular mechanisms can regulate the levels of transcripts carrying PTC. Our previous results showed that the nonsense-mediated mRNA decay (NMD) affects nonsense transcripts levels and modulates the response to readthrough treatment (Linde *et al*, 2007b). NMD is a translation-dependent surveillance mechanism conserved in all eukaryotic organisms, from yeast to humans (Culbertson, 1999). The NMD pathway detects and selectively degrades transcripts carrying PTCs, thus preventing the accumulation of truncated proteins that might be nonfunctional or deleterious due to dominant-negative or gain-of-function effects (Losson & Lacroute, 1979; Frischmeyer & Dietz, 1999; Durand & Lykke-Andersen, 2011). The NMD degrades not only aberrant transcripts from mutant genes, but is also an important post-transcriptional regulatory mechanism degrading normal transcripts from genes (encoding functional proteins) that harbor a normal stop codon in a context that elicits NMD (Mendell *et al*, 2004; Chang *et al*, 2007; Neu-Yilik & Kulozik, 2008). The role of the NMD mechanism in regulating gene expression and cell survival highlights the important biological function of NMD as an RNA surveillance mechanism under physiological conditions. Perturbation of the NMD mechanism may have major implications as a result of altered levels of many of its physiological substrates. Consistent with this, aberrant conditions such as malignant transformation as well as different cellular stress conditions involve NMD inhibition (Gardner, 2008, 2010; Wang *et al*, 2011a,b2011b). Importantly, under conditions of inefficient NMD, PTC-carrying transcripts tend to accumulate in the cell enabling a better response to readthrough agents (Linde *et al*, 2007b). In addition, the translation of these transcripts is expected to result in the accumulation of truncated proteins in the cell.

About one-third of all newly synthesized proteins in a cell are secretory and transmembrane proteins. The transcripts of these proteins are translocated into the endoplasmic reticulum (ER) lumen, where they are translated. The resulting proteins are folded, modified, and correctly assembled in the ER before being targeted to the rest of the endomembrane system, cell membrane or secretion, depending on their function and nature (Zhang & Kaufman, 2008). When misfolded proteins accumulate in the ER, the UPR is induced to restore cellular homeostasis by activating ER chaperones and foldases and by inhibiting translation of new substrates. Accumulation of unfolded proteins in the ER is the primary stress signal sensed in metazoans by three parallel pathways, in each branch a transmembrane protein: IRE1, PERK, or ATF6 senses the abnormal condition in the ER lumen or membrane and transmits the signal to the cytosol where a series of transcription factors carry information to the nucleus (Walter & Ron, 2011). The UPR coordinates an adaptive response to the stress and alters the cellular transcriptional and translational programs to enable cells to cope with the stressful condition and to resolve the protein-folding defect (Kaufman, 1999; Schroder, 2006; Brodsky, 2007; Zhang & Kaufman, 2008). Since UPR activation attenuates the general translation in the cell, it was recently shown to inhibit the NMD mechanism which is translational dependent (Wang *et al*, 2011b). However, this is expected to perturb the cellular homeostasis under ER stress conditions and increase the level of unfolded proteins.

In this study, we show that UPR activation regulates the levels of transcripts carrying disease-causing PTCs, and by this governs the response to readthrough treatment. Unbiased proteomic analyses showed that there are substantial differences in UPR activation between patients carrying PTCs that correlate with their response to readthrough treatment. Furthermore, an inverse relationship between the NMD and UPR processes was found in proteomic analyses of primary cells from patients and in various cell lines, suggesting a feedback loop between these two homeostatic mechanisms. Importantly, NMD inhibition together with UPR activation enhanced the response to readthrough treatment, highlighting the functional role of the NMD-UPR feedback loop. We further revealed and characterized a novel feedback loop between NMD and UPR that augments both the activation of the UPR and the attenuation of the NMD. Importantly, our study demonstrates that in order to maintain cellular homeostasis under ER stress, UPR factors are subjected to NMD, suggesting a novel, NMD-mediated mechanism by which the cell attempts to enhance the UPR activity in response to ER stress. Similarly, UPR itself attempts to activate the NMD by increasing the level of NMD factors, despite the natural downregulation caused by translation inhibition to resolve the ER protein load. Altogether, our study demonstrates the importance of the UPR and its regulatory network for genetic diseases caused by PTCs and for cell homeostasis under normal conditions.

## Results

### Differential UPR activation between two patients carrying the W1282X stop mutation in the CFTR gene

**Figure 1:**
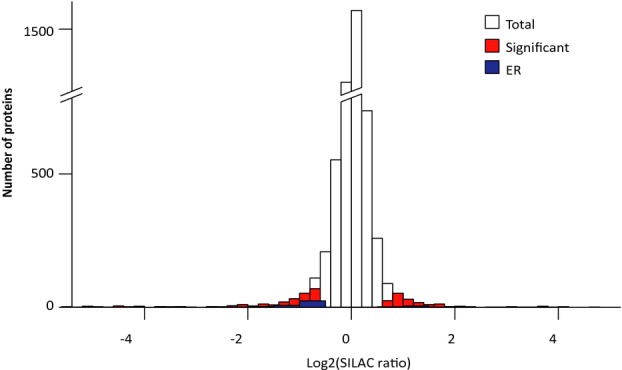
Differential UPR activation between two patients carrying the W1282X stop mutation in the CFTR gene. SILAC-based quantitative proteomic analysis of lymphoblastoid cell lines. A histogram of overall protein ratios shows high similarity between the patient-derived cells, with only 440 showing statistically significant differences (Significance < 0.05; Significant). Significantly different ER resident proteins (ER) are higher in the responsive CF patient.

Aiming to reveal cellular pathways which play a role in the inter-patient variability in the response to readthrough treatment, we performed an unbiased analysis of the proteome of two CF patients who are sisters, and whose parents are first-degree cousins, by high-resolution quantitative mass spectrometry (Cox & Mann, 2011). These sisters participated in a readthrough clinical trial (Wilschanski *et al*, 2003). Although both are homozygous for the CFTR W1282X allele, they differed in their response to the treatment, as measured by the normalization of the nasal potential difference (NPD), and in their CFTR transcript levels. Patient 6537 did not respond to the treatment and had a lower level of CFTR transcripts than her sister, patient 6538 (Wilschanski *et al*, 1995, 2000; Linde *et al*, 2007b). The proteome analyses were performed on two EBV-transformed lymphoblastoid cell lines derived from the two patients using the stable isotope labeling in cell culture (SILAC) technique for accurate quantification (Mann, 2006). Out of the 6000 proteins identified, only 440 proteins showed a statistically significant different level between the cell lines at a false discovery rate (FDR) of 0.05 (Fig 1). This very high similarity likely reflects the similar genetic background of the sisters as well as the precision of our proteomics quantification. Interestingly, more than 10% of the proteins that significantly changed (*n* = 43) are known ER resident proteins and can be activated by the UPR (Fig 1; examples are shown in Supplementary Table S1). Unbiased enrichment analysis using the Fisher's exact test showed enrichment of a small number of gene ontology categories and revealed a highly significant enrichment of ER proteins (FDR < 0.02; Supplementary Table S2). Altogether, the proteomic results raise the possibility that the UPR has a role in the regulation of readthrough treatment for patients carrying disease-causing PTCs.

### UPR activation enables CFTR and XLF function following readthrough treatment

**Figure 2:**
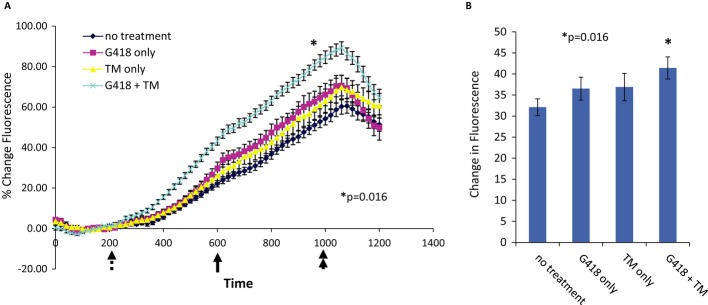
The effect of UPR activation on the CFTR chloride efflux following G418 treatment. A Halide efflux was measured using the SPQ fluorescence assay. Forskolin (20 μM) and genistein (50 μM) were added (solid arrow) to stimulate CFTR-dependent ion transport (rate of upward deflection tracks CFTR activity before and after pharmacologic stimulation). The findings indicate upregulation of CFTR following combined treatment with G418 and tunicamycin. Dotted arrow = addition of dequenching buffer; double arrow = addition of quenching buffer. B Summary data indicating stimulated efflux rate by treatment group. *P =* *0.016 by ANOVA;*n *=* *9–10 coverslips/condition, > 15 cells monitored per coverslip.

To assess the effect of UPR activation on the response to readthrough treatment, we studied two genetic diseases caused by PTCs. The first studied model system is CFP15a cells, a human epithelial cell line derived from a nasal polyp of a CF patient carrying the nonsense mutation W1282X and the splicing mutation 3849+10 kb C->T (which also leads to a PTC) in the CFTR gene (Linde *et al*, 2007b). We treated these cells with G418 (an aminoglycoside antibiotic used for readthrough) together with UPR activation and examined the functional changes in the cells. As a functional output, we examined the activity of the cAMP-activated chloride channel, which is encoded by the CFTR gene. Untreated CFP15a cells showed negligible chloride efflux (Fig 2, blue line). The readthrough agent G418 or the UPR activator tunicamycin (TM) alone had a slight (but not significant) increase in CFTR-mediated halide efflux (Fig 2, pink and yellow lines); however, the combined treatment resulted in considerable and significant improvement in halide efflux, implying the presence of functional CFTR channels in these cells (Fig 2, light blue line *P*-value = 0.016). Concomitant with these results, combined treatment of UPR activation together with readthrough treatment resulted in an increase in the level of CFTR proteins (Supplementary Fig S6).

These results show that the response to readthrough treatment in CFP15a cells was only possible following UPR activation.

**Figure 3:**
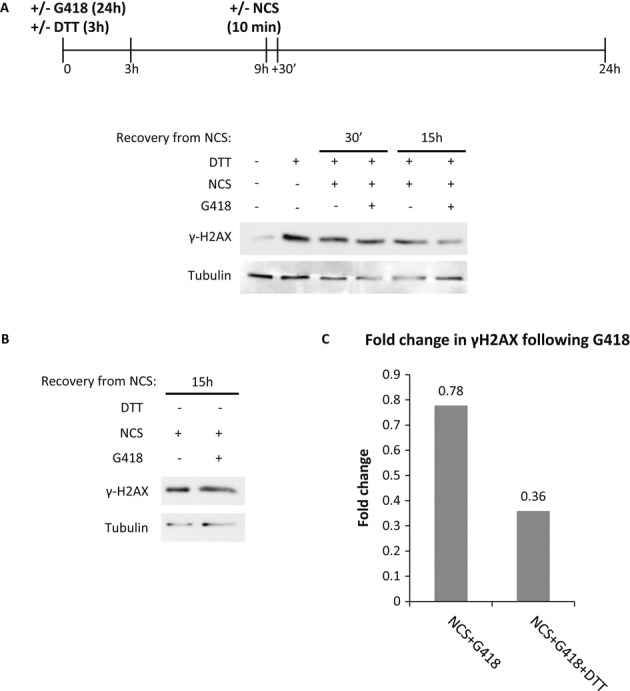
The effect of UPR activation on XLF function following G418 treatment. A, B P133 cells were grown in the presence or absence of 250 μg/ml G418 for 24 h with (A) or without (B) UPR induction by 10 mM DTT treatment for 3 h. Cells were allowed to recover for 6 h and then treated with 0.65 pg/μl NCS for 10 min. Cells were then harvested at the indicated times following the NCS wash. Protein extracts were prepared and analyzed by immunoblotting with anti-γH2AX and anti-tubulin antibodies. C Quantification of γH2AX levels normalized to tubulin.

Next we examined the effect of UPR activation following readthrough treatment in another disease model, a human fibroblast cell line (P133) derived from a patient with immunodeficiency with microcephaly, homozygous for the nonsense mutation R178X in the Cernunnos/XRCC4-like factor (XLF) gene (Buck *et al*, 2006). Cernunnos/XLF plays a role in the nonhomologous end joining (NHEJ) DNA damage repair, as a part of a protein complex together with DNA ligase IV and XRCC4 that ligate the two DNA ends. Fibroblasts from patients carrying Cernunnos/XLF mutations are radiosensitive and exhibit impaired DNA double-strand break (DSB) repair following IR or radiomimetic drug treatment (Ahnesorg *et al*, 2006; Buck *et al*, 2006). To evaluate the effect of UPR on XLF function, we analyzed the phosphorylation of histone H2AX on Ser-139 (a measure for the amount of double-stand breaks) following DSB induction with neocarzinostatin (NCS). Cernunnos/XLF mutated cell line, P133, was treated for 10 min with NCS and allowed to recover in regular media in the presence or absence of G418 and in the presence or absence of UPR activation by dithiothreitol (DTT) (Fig 3A). In P133 cells, the level of γH2AX (indicating the phosphorylated form) increased following NCS treatment and stayed high for 15 h of recovery (Fig 3B), indicating an impaired repair of DSBs in the absence of Cernunnos/XLF. G418 treatment by itself did not reduce the level of γH2AX (Fig 3B and C); however, UPR activation by DTT treatment together with readthrough by G418 resulted in reduced levels of γH2AX (Fig 3A, lane 6 in comparison with lane 5 and Fig 3C), indicating that the XLF protein was functional in these cells. These results indicate that UPR activation enables the response to readthrough treatment in P133 cells. We further examined the effect of UPR activation following readthrough in these cells by immunofluorescent analysis of γH2AX and 53BP1 colocalization, for the quantification of the DNA double-strand break repair (Supplementary Fig S1). Similar to the Western results, the immunofluorescent analysis also shows that UPR activation by DTT treatment together with readthrough by G418 significantly reduced levels of DNA double-strand breaks.

Altogether, the results from both the CFTR and XLF disease models indicate that indeed UPR activation plays an important role in modulating the response to readthrough treatments of cells carrying disease-causing PTC.

### UPR activation leads to upregulation of endogenous transcripts carrying disease-causing PTCs

**Figure 4:**
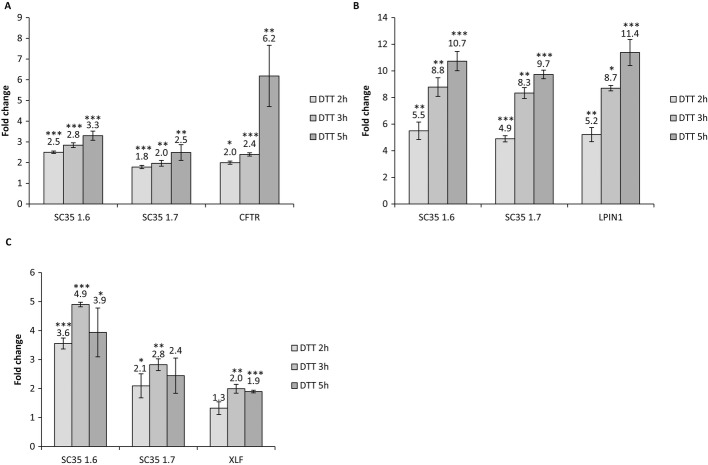
UPR activation leads to upregulation of transcripts carrying disease-causing PTCs. A–C CFP15a (A), LPIN1 (B), and P133 (C) cells were treated with DTT (10 mM) for 2, 3, and 5 h. The level of SC35 1.6, SC35 1.7 and the level of CFTR or LPIN1 or XLF1 transcripts were measured by RT-qPCR. The values shown are the average fold change (mean ± s.e.m.) from at least three independent experiments relative to nontreated cells. Values were normalized against transcripts of RNA polymerase II gene. Statistical analysis was performed using Student's *t*-test (one-tailed, paired). *P <* *0.05, **P <* *0.01, ***P <* *0.001.

The level of the transcripts carrying PTCs is a limiting factor in response to readthrough treatments, such that response is found only in patients with relatively high levels of transcripts (Linde *et al*, 2007b; Kerem *et al*, 2008). We therefore examined the effect of UPR activation on the level of transcripts carrying disease-causing PTCs. To measure this effect, we analyzed cell lines derived from patients carrying different disease-causing PTCs: the CFP15a carrying a PTC in the CFTR gene (Linde *et al*, 2007b); P133, carrying PTC in XRCC4-like factor (XLF) gene, as described above (Buck *et al*, 2006); and LPIN1, human primary fibroblasts derived from a patient with myoglobinuria, homozygous to the nonsense mutation E215X in the LPIN1 gene (Zeharia *et al*, 2008). UPR activation by DTT treatment led to significant increases in the levels of CFTR, XLF1, and LPIN1 transcripts (Fig 4A–C). These results highlight the dual effect of UPR activation in regulating both transcripts and proteins levels and shed a light on the mechanism by which UPR activation regulates the response to readthrough treatment. It is important to note that G418 had no effect on the level CFTR and XLF transcripts (Supplementary Fig S2A), indicating that G418 by itself did not contribute to the functional correction of the proteins.

We hypothesized that the effect of UPR on the transcript levels is mediated through NMD regulation. In agreement with Wang *et al* (2011b), we show that activation of UPR inhibits the NMD by showing significant increases in the levels of known NMD substrates SC35 1.6 and SC35 1.7 (Sureau *et al*, 2001) (Fig 4, Supplementary Fig S3). UPR activation by TM in CFP15a had the same effect on the transcript levels of CFTR and known NMD substrates (Supplementary Fig S2B), supporting the CFTR functional results presented in Fig 2.

The effect of NMD inhibition on the level of known NMD substrates and transcripts carrying disease-causing PTCs was further analyzed in CFP15a, P133, and LPIN1 cells. As can be seen in Supplementary Fig S2C, the effect of NMD inhibition on the transcript levels was comparable to that observed following UPR activation (Fig 4). Hence, the UPR mechanism plays an important role in modulating the response to readthrough treatments by allowing transcripts carrying PTC to accumulate in the cell due to NMD attenuation. In order to verify that the upregulation of known NMD transcripts as well as transcripts carrying disease-causing PTCs is specific for NMD inhibition following UPR activation, and is not a general effect on the cells, we have performed analysis of the transcript levels of a large number of genes. As can be seen in Supplementary Fig S2D, DTT treatment did not lead to any change in their level, indicating that the increased level of NMD substrates results from NMD inhibition due to UPR activation and is not a general effect of DTT treatment in the cells.

### NMD inhibition activates the PERK-peIF2α UPR branch

**Figure 5:**
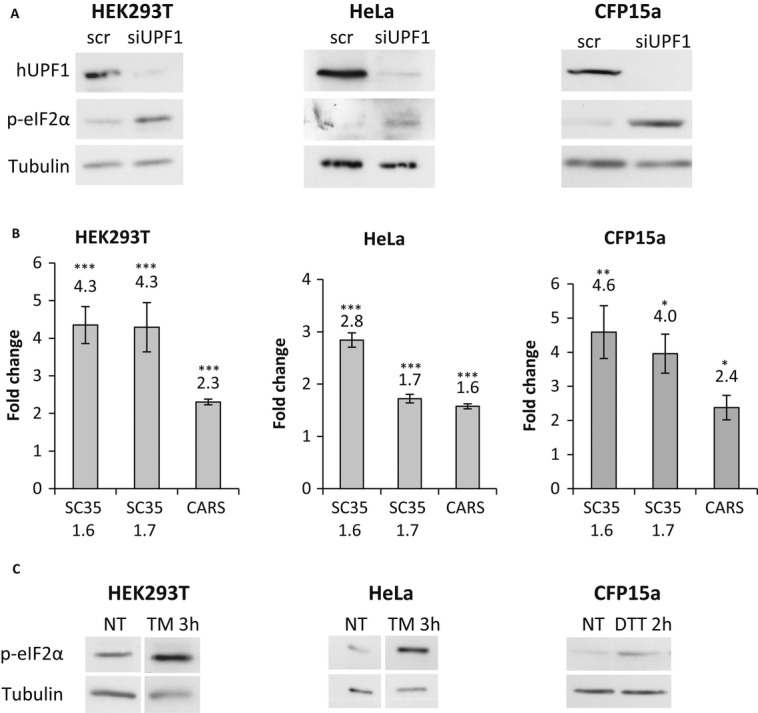
NMD inhibition activates the PERK-peIF2α UPR branch. A, B HEK293T, HeLa, and CF15a cells were transfected with siRNA against hUPF1 or nonspecific control siRNA (scr) for 48 or 72 h. (A) Protein extracts were prepared and analyzed by immunoblotting with anti-hUPF1, anti-tubulin, and anti-peIF2α antibodies. (B) Levels of SC35 1.6, SC35 1.7, and CARS transcripts were measured by RT-qPCR. The values shown are the average fold change (mean ± s.e.m.) from at least three independent experiments relative to nontreated cells or cells transfected with a nonspecific control siRNA. Values were normalized against transcripts of RNA polymerase II gene (HEK293T and HeLa) or GAPDH (CFP15a). The values shown are the average fold change (mean ± s.e.m.) from at least three independent experiments relative to nontreated cells. Values were normalized against transcripts of RNA polymerase II gene. Statistical analysis was performed using Student's *t*-test (one-tailed, paired). *P <* *0.05, **P <* *0.01, ***P <* *0.001. C HEK293T, HeLa, and CFP15a cells were treated with TM (25 μg/ml) for 3 h or DTT (10 mM) for 2 h. Protein extracts were prepared and analyzed by immunoblotting with anti-tubulin and anti-peIF2α antibodies.

The UPR and NMD mechanisms have an important role in modulating the response to readthrough treatment. Importantly, both mechanisms have a crucial role in maintaining homeostasis under physiological and environmental conditions. Although it is no wonder that translational attenuation causes a decrease in NMD, we speculated that this decrease would cause the accumulation of NMD physiologic transcripts and more truncated proteins. If these proteins are transmembrane or secretory, then NMD inhibition should, in return, lead to UPR activation. Based on Gene Ontology (Ashburner *et al*, 2000), we created a list of approximately 6000 genes encoding proteins that are translated and processed in the ER (ER substrates). Using unbiased expression data, we identified ER substrates which were upregulated following hUPF1 downregulation in HeLa (Mendell *et al*, 2004) or HEK293T cells (our expression arrays). Among the upregulated transcripts in HeLa cells (*n* = 244) and HEK293 (*n* = 226), multiple transcripts (95 and 44, respectively) corresponded to ER substrates, indicating that there is a large number of transcripts processed in the ER that are regulated by the NMD mechanism. These results strengthen the notion that indeed under conditions of inefficient NMD, the UPR will be activated. Recently, Sakaki *et al* (2012) showed that NMD inhibition indeed leads to activation of the UPR through the IRE1α-spliced XBP1 branch (Supplementary Fig S4) as detected by a significant increase in the level of spliced XBP1 transcripts similar to the increase found following mild DTT-induced stress. Since NMD attenuation is induced by the PERK-peIF2α UPR branch, we examined whether NMD inhibition activates also this UPR branch. For this purpose, we inhibited the NMD pathway using siRNA directed against one of its essential factors, hUPF1 (Mendell *et al*, 2002). The analysis was performed in two epithelial cell lines, HeLa and HEK293T, and in a nasal epithelial cell line, CFP15a (Linde *et al*, 2007b). Western blot analysis showed a significant sequence-specific downregulation of *UPF1,* 48 h after transfection in HeLa cells and 72 h in HEK293 and CFP15a cells (Fig 5A). Using RT-qPCR, we could show that *UPF1* downregulation causes an increase in the levels of several known physiological NMD substrates, SC351.6, SC351.7, and CARS (Sureau *et al*, 2001; Mendell *et al*, 2004) (Fig 5B).

Next we investigated the effect of NMD inhibition on UPR activation by analyzing the phosphorylation of eIF2α (Shen *et al*, 2004; Kohno, 2007). Using Western blot analysis, we detected a substantial increase in the level of phosphorylated eIF2α in all three tested cell lines (Fig 5A and Supplementary Fig S5) in a manner similar to control cells treated with the known UPR activators, TM or DTT (Fig 5C and Supplementary Fig S5) (Lemin *et al*, 2007). These results indicate that NMD inhibition indeed leads to activation of the PERK-peIF2α UPR branch. Activation of the PERK-peIF2α UPR branch following NMD inhibition demonstrates an important crosstalk between these two regulatory pathways, implying a mechanistic link between them.

### A feedback loop between the NMD mechanism and the ER stress response

**Figure 6:**
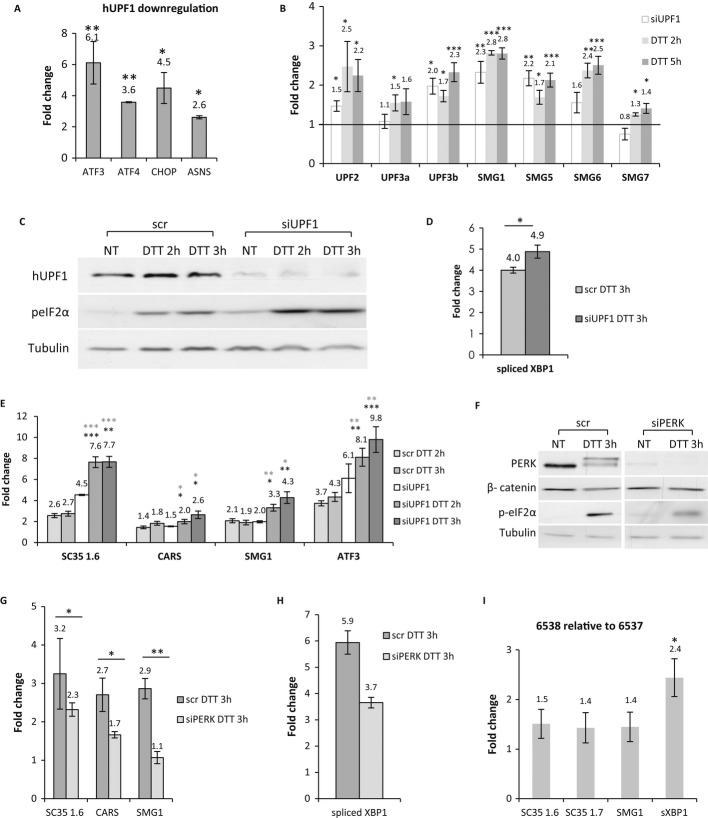
A positive feedback loop between the NMD and the UPR mechanisms. A HEK293T cells were transfected with siRNA against hUPF1 or nonspecific control siRNA (scr) for 72 h. Levels of ATF3, ATF4, CHOP, and ASNS transcripts were measured by RT-qPCR. The values shown are the average fold change (mean ± s.e.m.) from at least three independent experiments relative to nontreated cells. Values were normalized against transcripts of RNA polymerase II gene. Statistical analysis was performed using Student's *t*-test (one-tailed, paired). *P <* *0.05, **P <* *0.01, ***P <* *0.001. B HeLa cells were treated with DTT (10 mM) for 2 and 5 h or transfected with siRNA against hUPF1 or nonspecific control siRNA (scr) for 48 h. The levels of the NMD factors transcripts were measured by RT-qPCR. Data were quantified and statistically analyzed as in (A). C–E HEK293T cells were transfected with siRNA against hUPF1 or nonspecific control siRNA (scr) for 72 h with or without DTT treatment (10 mM) for 2 or 3 h. (C) Protein extracts were prepared and analyzed by immunoblotting with anti-hUPF1, anti-tubulin, and anti-peIF2α antibodies. (D) Levels of spliced XBP1 and (E) SC35 1.6, CARS, SMG1, and ATF3 transcripts were measured by RT-qPCR. Data were quantified and statistically analyzed as in (A). All the changes following UPR activation by DTT and UPF1 downregulation were significantly higher compared to nontreated or scr cells. Black asterisks: scr DTT/siUPF1 DTT. Gray asterisks: siUPF1 DTT/siUPF1. F–H HEK293T cells were transfected with siRNAs against PERK or nonspecific control siRNA (scr) for 72 h with or without DTT treatment (10 mM) for 3 h. (F) Protein extracts were prepared and analyzed by immunoblotting with anti-PERK, anti-tubulin, anti-β catenin, and anti-peIF2α antibodies. (G) Levels of SC35 1.6, CARS, and SMG1. (H) Levels of spliced XBP1 (*P*-value = 0.05). I SC35 1.6, SC35 1.7, SMG1 and sXBP1 transcripts were measured by RT-qPCR. Data represent the ratio between 6538 and 6537 lymphoblastoid cell lines. Data were quantified and statistically analyzed as in (A).

We postulated that one way of creating a link between NMD and UPR is by subjecting specific UPR factors to NMD regulation. We therefore analyzed whether the transcript levels of key UPR factors increase during NMD inhibition. For this, we inhibited the NMD pathway using siRNA directed against UPF1 and analyzed the expression levels of four known UPR factors: ASNS (Gjymishka *et al*, 2009), ATF3, ATF4, and CHOP (Fribley *et al*, 2011). We found that NMD inhibition led to upregulation of all these transcripts (Fig 6A). Thus, NMD modulates the expression levels of UPR factors.

**Figure 7:**
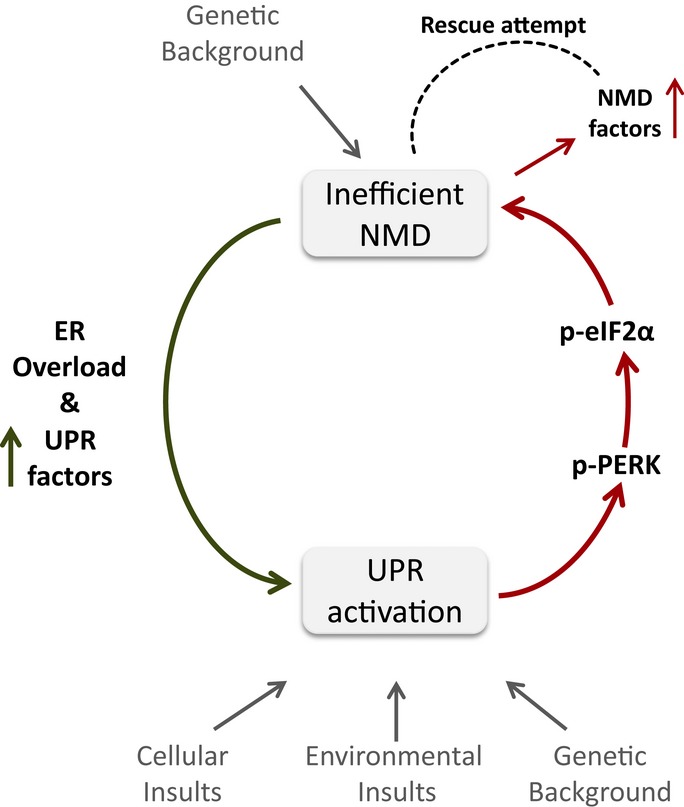
Feedback-loop regulatory network between the NMD and UPR pathways augments both the activation of the UPR and the attenuation of NMD and confers homeostasis under genetic, environmental, and/or cellular insults.

A gene may be upregulated following UPF1 knockdown either because it is a direct NMD target, or due to secondary effects. In order to test whether our four genes are likely to be *bona fide* NMD targets, we looked whether they harbor known NMD-triggering features (Schweingruber *et al*, 2013): long 3′ untranslated region (3′UTR), an intron more than 55 bases downstream of the stop codon (deep intron), and an upstream open reading frame (uORF). We have found that ASNS (ENST00000454046) harbors a deep intron, ATF3 (ENST00000366987) has long 3′UTR, and three isoforms of ATF4 have uORFs (ENST00000337304, ENST00000396680, and ENST00000404241), whereas for the CHOP gene we could not detect any of the known NMD-triggering classical features. These results indicate that upregulation of ATF3, ATF4, and ASNS transcripts (observed after UPF1 knockdown) is likely to result from a direct NMD effect. This reveals a component of the NMD-UPR feedback loop where, under ER stress conditions, NMD attenuation increases the levels of these factors in an attempt to improve the ability of the cells to resolve the ER protein load (Fig 7).

Downregulation of NMD upon UPR activation is expected to increase ER load due to increased levels of misfolded proteins. While activation of NMD is expected to alleviate ER stress, our results clearly show that UPR activation inhibits rather than activates NMD. Recently, it has been shown that the efficiency of NMD is tightly regulated by a negative feedback in which low NMD activity induces an increase in the transcript levels of NMD factors (Chan *et al*, 2007; Yepiskoposyan *et al*, 2011). This prompted us to investigate whether ER stress conditions can affect this NMD autoregulation mechanism and upregulate the mRNAs of NMD factors. Indeed, ER stress induced by DTT significantly increased the mRNA levels of the NMD autoregulated factors UPF2, UPF3a, UPF3b, SMG1, SMG5, SMG6, and SMG7 (Fig 6B). This increase was similar to that observed following direct NMD inhibition by siRNA directed against hUPF1 (Fig 6B). Two of the NMD factors, hUPF3a and SMG7, were upregulated following UPR activation but not under hUPF1 downregulation (Fig 6B), suggesting that the UPR has a broader effect on the NMD autoregulated factors. Overall, our results show an inverse relationship between NMD factors transcript levels and the NMD activity. Moreover, these results demonstrate that cells have evolved mechanisms, which attempt to increase NMD function under conditions of folding stress, despite a global reduction in translation.

We have shown that UPR activation inhibits NMD and that NMD inhibition activates UPR, thereby suggesting that there may be a feedback loop between these two cellular homeostatic mechanisms. To investigate this, we tested whether NMD inhibition augments the response to induction of ER stress. We first analyzed the levels of phospho-eIF2α and spliced XBP1 in HEK293T cells treated with DTT with or without hUPF1 downregulation. Both phospho-eIF2α and spliced XBP1 increased significantly in the combined treatment in comparison with DTT treatment or hUPF1 downregulation alone (Fig 6C and D, Supplementary Fig S4). Next we analyzed the combined effect of NMD inhibition and UPR activation on the levels of physiological substrates of NMD. In agreement with the previous results, we found a significant increase in the transcript levels in the combined treatment as would be expected from synergistically acting pathways (Fig 6E). These results strongly support the existence of a feedback loop between the two homeostatic mechanisms, NMD and UPR (Fig 7).

To further test the NMD-UPR feedback loop, we interfered with the UPR-mediated NMD inhibition. For this, we downregulated PERK expression and analyzed the effect of DTT treatment on the IRE1-spliced XBP1 UPR branch. PERK phosphorylates eIF2α to attenuate translation initiation and thus is responsible for the NMD attenuation. As a control, we confirmed that PERK downregulation results in reduced eIF2α phosphorylation and NMD inhibition as previously described (Wang *et al*, 2011b) (Fig 6F). Most importantly, PERK downregulation reduced NMD inhibition in response to DTT treatment (Fig 6G). Interestingly, in these same cells, UPR activation, through the IRE1 branch, was also reduced despite the fact that one might expect compensation during the loss of one signaling arm (Fig 6H). These results suggest that proper function of NMD reduces the ER stress further supporting the feedback loop between NMD and UPR mechanisms (Fig 7).

Finally, we analyzed the NMD-UPR feedback loop in our lymphoblastoid cell lines derived from the two sisters (Fig 1). For this, we have analyzed the transcript level of spliced-XBP1 and found an higher level of this UPR factor in the cell line derived from the responding sister (patient 6538) compared to the level in the nonresponding sister (patient 6537) (Fig 6I). This result fully supports the differences found in the proteomic analyses between these cell lines (Fig 1). We further identified higher transcript levels of known NMD substrates (SC35 1.6, SC35 1.7, and SMG1) in the cells from the responding sister (Fig 6I). Altogether, the analyses in the patient cell lines support the feedback-loop model.

### A negative correlation between NMD and UPR factors *in vivo*

**Figure 8:**
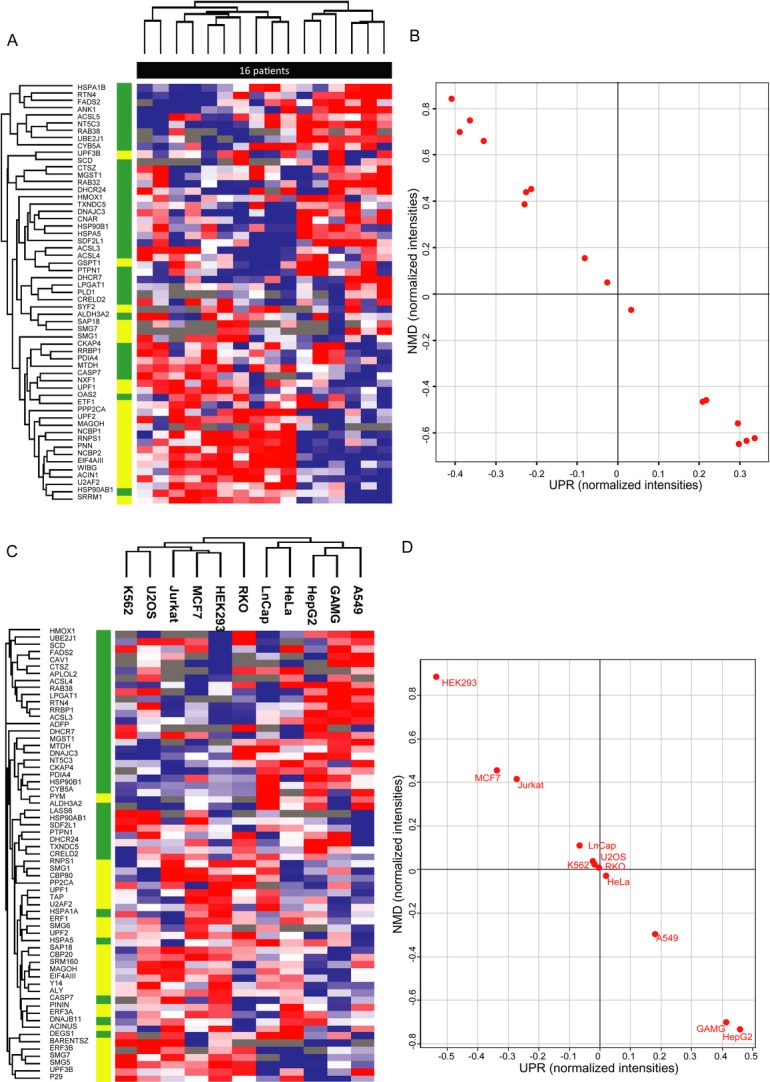
A negative correlation between NMD and UPR factors. A–D Hierarchical clustering (A, C) and scatter plots (B, D) of 58 NMD and UPR factors in 16 primary lymphocytes from CF patients (A, B) and commonly used cell lines (Geiger *et al*, 2012) (C, D). For the scatter plots, we averaged the normalized ratio values (primary lymphocytes) or normalized intensities (cell lines) of each group of proteins (NMD or UPR).

The feedback loop described above is suggested as a general cellular homeostatic mechanism. To examine its generality, we first expanded our SILAC analysis to 16 samples of lymphocytes isolated from blood samples of CF patients, all carrying a stop mutation in the CFTR gene. The patients are currently participating in a phase IIb clinical trial using the readthrough drug ataluren (PTC124), which is expected to be completed in 2014. We examined the levels of the core NMD factors as well as ER resident proteins that are activated under conditions of UPR activation whose level differed between the CF sisters (Fig 1 and Supplementary Table S3). The results show a statistically significant negative correlation between the levels of NMD and UPR factors (Spearman's rank correlation = −0.97) (Fig 8B). As can be seen in Fig 8, in patients that have high levels of NMD factors, the levels of UPR factors are low and vice versa (Fig 8). These results highlight the inverse relationship between the two processes both *in vitro* (Fig 7) and *in vivo*.

We hypothesized that this relationship is not limited to disease-related cells harboring PTC mutations, but is rather a more general regulatory mechanisms of gene expression. We therefore performed a similar analysis on a recently published large-scale proteomic dataset of eleven commonly used cell lines K562, RKO Jurkat, HEK293, LnCap, MCF7, U2Os, HepG2, GAGM, and A549 (Geiger *et al*, 2012). In agreement with the CF samples, a perfect negative correlation between the levels of NMD and UPR factors was found (Fig 8C and D; Spearman's rank correlation = −1). Altogether, the proteomic results show a clear negative correlation between NMD and UPR, indicating that there is a mechanistic link between them. This feedback loop has a role under physiological conditions and in human genetic diseases resulting from PTCs.

### NMD inhibition together with UPR activation enhances the response to readthrough treatment

**Figure 9:**
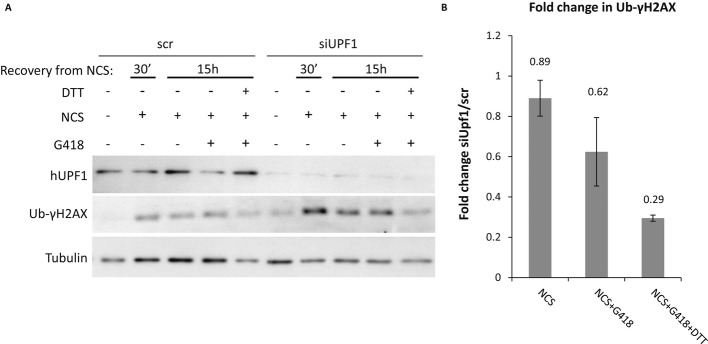
NMD inhibition together with UPR activation enhances the response to readthrough treatment. P133 cells were transfected with siRNA against hUPF1 or nonspecific control siRNA (scr) for 72 h. DTT, G418, and NCS treatments were performed as described in Fig 3A. A Protein extracts were prepared and analyzed by immunoblotting with anti-hUPF1, anti-γH2AX, and anti-tubulin antibodies. B Quantification of Ub-γH2AX levels normalized to tubulin. All siUPF1 samples were normalized according to the nontreated siUPF1. Data represent the fold change of Ub-γH2AX in siUPF1 compared to scr, for each indicated treatment. Quantification is an average of two experiments.

The NMD-UPR feedback-loop mechanism predicts that NMD inhibition together with UPR activation enhances the response to readthrough treatment, in cells carrying disease-causing PTCs, compared to the effect of each treatment alone (NMD inhibition or UPR activation). To evaluate the effect of UPR activation together with NMD inhibition on XLF function following readthrough, we analyzed the ability of P133 cells (carrying a PTC in the XLF gene) to repair DNA DSBs, reflecting the XLF function, by analyzing the levels of ubiquitylated γH2AX (UB-γH2AX). Ubiquitylation of γH2AX is an important event in DNA damage response (Messick & Greenberg, 2009; Sharma *et al*, 2014) that facilitates the recruitment of DDR factors and might affect chromatin structure, leading to repair of the damage (Bergink & Jentsch, 2009; Messick & Greenberg, 2009). P133 cells transfected with siRNA directed against hUPF1 (for NMD inhibition) or scrambled control were treated for 10 min with NCS and allowed to recover in a regular media in the presence or absence of G418 and in the presence or absence of UPR activation by DTT. We have analyzed the repair efficiency of DSBs in UPF1 KD cells compared to control cells for each treatment (Fig 9A). The levels of Ub-γH2AX increased following NCS treatment and stayed high for 15 h of recovery (lanes 2 and 3, 7, and 8). G418 treatment by itself did not repair the DSBs (compare lane 4 to lane 3). Since UPF1 KD caused DSBs (compare lane 6 to lane 1), the level of Ub-γH2AX in all KD samples was normalized according to nontreated UPF1 KD. UPF1 KD together with readthrough by G418 resulted in DSBs repair. Importantly, the most efficient repair by readthrough was observed following both activation of UPR and NMD inhibition (Fig 9B). These results show an improved restoration of the XLF function following readthrough under combined NMD and UPR treatments and highlight the functional role of the NMD-UPR feedback loop in modulating readthrough treatment.

## Discussion

Here, we show that UPR governs the response to readthrough treatment (Figs 4). Proteome analyses show a significant negative correlation between the UPR and NMD (Fig 8), suggesting a feedback loop between these two homeostatic mechanisms. Importantly, combined NMD inhibition and UPR activation enhanced the response to readthrough treatment (Fig 9), shedding a new light on the functional role of the NMD-UPR feedback loop. We further uncovered and characterized this novel negative feedback loop, which augments both the activation of the UPR and the inhibition of the NMD. Altogether, these results highlight the importance of the NMD and UPR mechanisms to maintain homeostasis under normal conditions and in genetic diseases caused by PTCs.

Variability in NMD efficiency is found in cell lines, tissues, and among individuals, suggesting that the efficiency of NMD is an inherent feature of cells (Holbrook *et al*, 2004; Linde *et al*, 2007a) that can be modulated by genetic traits (sequence variations) (Tarpey *et al*, 2007), environmental conditions (hypoxia, amino acid starvation, etc.) (Wang *et al*, 2011b), or aberrant cellular regulation (tumorigenicity) (Wang *et al*, 2011a). Our results showing a feedback loop between NMD and the UPR suggest that either the variability in UPR activation results from variability in NMD efficiency or vice versa.

Both NMD and UPR factors are regulated by post-translational modifications, including phosphorylation (Chang *et al*, 2007; Ron & Walter, 2007; Popp & Maquat, 2013). In the future, analysis of post-translational modifications will add another layer of information about the regulation of these processes.

About one-third of all monogenic inherited diseases result from PTCs, which are subjected to NMD (Kuzmiak & Maquat, 2006). One of the approaches for the treatment of patients carrying PTCs is readthrough by drugs affecting the ribosome decoding site, such as aminoglycoside antibiotics (Linde & Kerem, 2008) and more recently ataluren (PTC124) (Kerem *et al*, 2008; Sermet-Gaudelus *et al*, 2010). Readthrough studies often reveal unexplained variable responses to the treatment (Barton-Davis *et al*, 1999; Wilschanski *et al*, 2000, 2003; Wagner *et al*, 2001; Dunant *et al*, 2003; Politano *et al*, 2003; Clancy *et al*, 2007; Kerem *et al*, 2008). Our results show the importance of UPR in regulating the response to readthrough treatment in CF patients (Fig 1, patient 6538 relative to patient 6537). We have previously shown that the level of nonsense transcripts is a limiting factor in the response to readthrough treatments (Linde *et al*, 2007b; Kerem *et al*, 2008). Here, we found that the expression level of transcripts carrying disease-causing PTCs can be modulated by UPR activation (Fig 4), supporting our basic finding that activation of the UPR leads to downregulation of NMD activity (Fig 4 and Supplementary Fig S3) and shedding light on the cellular mechanism by which UPR activation governs the response to readthrough treatment (Figs 2 and 3). UPR induction results in translation attenuation leading to NMD inhibition; therefore, UPR induction provides a mechanism by which transcripts carrying PTC can be rescued and serve as a template for readthrough treatment. It is important to note that in the two disease models: CFTR and XLF, G418 alone did not have a significant effect on the protein function, indicating that upregulating the level of the PTC-bearing transcripts is required for a successful readthrough treatment. These results prove that UPR is a modifier of the response to readthrough treatment and therefore show for the first time that UPR plays a role in modulating the response to a mutation-specific treatment. It is interesting to note that several studies have shown upregulation of transcripts carrying PTCs following readthrough treatments using suppressor tRNA (Sako *et al*, 2006), G418 (Correa-Cerro *et al*, 2005), or amlexanox (Gonzalez-Hilarion *et al*, 2012) in mammalian cells. In these cases, the readthrough agents also inhibit the degradation of transcripts carrying PTCs.

Many human diseases are associated with ER stress and UPR activation (Zhang & Kaufman, 2008; Austin, 2009). For example, UPR is triggered by infection and inflammation in CF airway epithelial cells due to intensive synthesis of inflammatory mediators and defensive factors which results in an increased flux of these newly synthesized proteins into the ER (Bartoszewski *et al*, 2008; Martino *et al*, 2009; Ribeiro & Boucher, 2010). Interestingly, cancer progression is identified with rapidly proliferating cells which require increased levels of protein synthesis and processing (Luo & Lee, 2013), resulting in activation of the UPR. In the context of readthrough therapy, an inefficient NMD, as an inherited character of the cell or as a result of UPR activation, is expected to lead to upregulation of nonsense transcripts and by that to improve the response to the treatment. Strategies designed to induce the UPR locally may therefore improve the therapeutic benefits afforded by readthrough therapy. Moreover, we envision that in the future, screens for patients with high UPR activation will identify patients with higher potential to respond to readthrough treatment.

It is interesting to note that UPR activation by itself might have the potential to improve the CFTR function of the truncated W1282X protein. As seen in Fig 2, there is a slight (although not significant) increase in CFTR function following TM treatment alone. Recently, it has been shown that CFTR proteins truncated early in the nucleotide binding domain 2 (up to aa 1248) are exported from the ER, escape the ER quality control, and form a stable and functional chloride channel that is stable at the cell surface (Cui *et al*, 2007). Furthermore, HeLa cells stably expressing the W1282X CFTR cDNA show partial restoration of the CFTR function by enhanced expression of CFTR proteins carrying this nonsense mutations (Rowe *et al*, 2007). Therefore, imbalance between the protein load in the ER (as a result of overload induced by cDNA expression or as a result of impaired protein folding due to TM treatment) and the capacity of the ER enables the escape of truncated misfolded protein from the ER quality control. In cases in which the truncated protein has residual activity, as in case of the truncated W1282X CFTR protein, this will lead to a better CFTR function. These results suggest that patients harboring the W1282X mutation are susceptible to CFTR rescue by enhanced expression and localization of the CFTR truncated proteins when sufficient levels are localized to the plasma membrane due to UPR induction. Hence, UPR induction may be a viable way to provide therapeutic benefit for some diseases, either by increasing the level of partially functional truncated proteins or in combination with readthrough therapy to restore full-length functional proteins. We propose that in disorders like CF, where as little as 10–15% of normal channel function is sufficient for clinical rescue of the disease phenotype (Chu *et al*, 1992), mild UPR induction together with readthrough treatment can serve as a novel therapeutic approach.

The proteomic results clearly show a negative correlation between NMD and UPR factors among patients and cell lines. The entire proteomic results reflect the novel feedback loop between the NMD and UPR characterized in this study (Fig 7). We have further shed light on the mechanism activating the UPR by discovering that NMD inhibition modulates the transcription levels of a substantial number of ER substrates. This suggests that NMD normally has a role in regulating the protein load in the ER as part of the cellular homeostasis. Furthermore, we find that PERK-peIF2α is a driving force of the NMD-UPR feedback loop (Figs 5 and 6G).

Another mechanistic aspect of the NMD-UPR feedback loop emerging from our data is that UPR factors are subjected to NMD (Fig 6A). This suggests a novel, NMD-mediated mechanism by which the cell attempts to enhance the UPR activity in response to ER stress. Similarly, UPR can rescue the inherent propensity of translation inhibition to reduce NMD by utilizing the newly identified NMD homeostasis loop (Chan *et al*, 2007; Yepiskoposyan *et al*, 2011). Under conditions of general translation attenuation, NMD inhibition results in upregulation of NMD factors transcripts, in an attempt to improve NMD function (Fig 6B). The feedback loop between NMD and UPR implies that the differential sensitivity of cells to activate the UPR may also be the basis of variability in NMD efficiency. Similarly, variability in NMD efficiency may result from variable sensitivity to UPR activation.

Importantly, NMD inhibition together with UPR activation enhanced the response to readthrough treatment (Fig 9) highlighting the functional role of the NMD-UPR feedback loop in therapeutic approaches aimed to promote translational readthrough.

Altogether, our study provides new insights into the cellular mechanisms and the interplay between them in regulating readthrough and may enable the development of novel drugs aimed to improve the response to readthrough therapies for many human genetic diseases caused by PTCs.

## Materials and Methods

### Cell culture and treatments

HeLa and HEK239T cells were grown in Dulbecco's modified Eagle's medium supplemented with 10% fetal calf serum (FCS). CFP15b cells described previously (Linde *et al*, 2007b) were grown in bronchial epithelial cell basal medium. LPIN1 primary fibroblasts were grown in Dulbecco's modified Eagle's medium supplemented with 15% fetal calf serum (FCS). P133 cells described previously (Buck *et al*, 2006) were grown in RPMI medium supplemented with 10% fetal calf serum. 6537 and 6538 cell lines are EBV-immortalized B cells derived from CF patients and these cell lines were grown in RPMI medium supplemented with 15% fetal calf serum.

UPR induction was performed using 10 mM dithiothreitol (DTT) or 25 μg/ml tunicamycin (TM) for the indicated times.

### RNA interference

Short-interfering RNA (siRNA) directed against UPF1 (Dharmacon) were described by Mendell *et al*, (2002). Three siRNA sequences directed against PERK (Invitrogen) were used: 5′-GGCAGUGGAGUUUCUUCACAGUAAA-3′, 5′-CACCAGUAGCAA AUCUUCUUCUGAA-3′, and 5′-CAGAUGGAGAGAGUCAGGACCUUAA-3′.

Nonspecific control oligo (IDT) was used as control siRNA. Oligofectamine (Invitrogen) was used for transfection. Cells were harvested 48 h in HeLa cells, 72 h in HEK293T or CFP15a cells and 96 h in LPIN1 cells after transfection.

### RNA analysis

Total RNA was extracted using the RNeasy extraction kit (QIAGEN). RNA-less and reverse transcriptase-less reactions were used as controls. Complementary DNA (cDNA) synthesis was performed using the High Capacity cDNA Reverse Transcription kit (Applied Biosystems). Real-time PCR was subsequently performed in ABI 7500 using a Power SYBR green PCR master Mix (Applied Biosystems).The expression level was normalized to the transcript levels of RNA polymerase II gene and GAPDH. Specific primers for these PCRs were designed using the Primer Express software. For statistical analysis, Student's *t*-test was used. The actual *P*-values are summarized in Supplementary Table S5. The sequences of all used primers are described in Supplementary Table S4.

### Western blot analysis

10% and 6% polyacrylamide gels were used for protein separation. The gel was transferred to a nitrocellulose membrane, and antibody hybridization and chemiluminescence were performed according to the standard procedures. The primary antibodies used in this analysis were rabbit anti-hUPF1, kindly provided by Andreas E. Kulozik (University of Heidelberg, Heidelberg, Germany), rabbit anti-phosphor-eIF2α (Cell Signaling), rabbit anti-PERK (Cell Signaling), mouse anti-CFTR (596) kindly provided by John R Riordan, mouse anti-tubulin (SIGMA) and mouse anti-β-catenin (BD Transduction Laboratories). HRP-conjugated anti-rabbit and anti-mouse secondary antibodies were obtained from Jackson ImmunoResearch Laboratories.

### Immunofluorescence

P133 cells were fixed in 3.7% formaldehyde/PBS for 10 min, permeabilized with 0.5% Triton/PBS, and blocked with 10% FCS/PBS. The primary antibodies used were mouse anti-γH2AX (Upstate Biotechnology) and rabbit anti-53BP1 (Bethyl Laboratories). Appropriate Cy3- and Cy5-conjugated secondary antibodies were added (Jackson ImmunoResearch Laboratories). Images were taken with a Bio-Rad confocal microscope, and analysis was performed using ImageJ. Colocalization of both signals was considered as a DSB foci. Foci analysis was performed from at least 45 nuclei for each condition.

### Microarray data analysis

RNA was extracted from HEK293T cells transfected with siRNA directed against UPF1 or nonspecific control oligo (scr), using the RNeasy extraction kit (QIAGEN). Hybridization to GeneChip Human Gene 1.0 ST expression arrays, washing and scanning were performed according to the manufacturer's protocol (Affymetrix). Arrays were analyzed using RMA probeset condensation algorithm (Expression Console, Affymetrix). Genes were considered differentially expressed when the average change was over 1.5-folds. Analysis of published datasets of gene expression following hUPF1 downregulation in HeLa (GSM29530, GSM29531, GSM29532, and GSM29534) was performed as described previously (Mendell *et al*, 2004). ER substrates list was based on the dataset of The Gene Ontology Consortium (Ashburner *et al*, 2000) using: GO:0005886, GO:0005887, GO:0031226, GO:0005615, GO:0005578, GO:0044420, GO:0005794, GO:0030130, GO:0005768, and GO:0005764. We exclude genes which appear at: GO:0030130, GO:0010787, GO:0010788, GO:0010009, GO:0032419.

### SILAC labeling and sample preparation for MS analysis

6537 or 6538 cells were SILAC-labeled by culturing them for 14 doublings in medium deprived of lysine and arginine and supplemented with heavy forms of these amino acids: ^13^C_6_^15^N_4_-arginine (Arg-10) and ^13^C_6_^15^N_2_-lysine (Lys-8) or with the light amino acids. Medium was supplemented with dialyzed serum to eliminate the light amino acids from the serum. Cells were harvested in lysis buffer consisting of 4% SDS, 100 mM Tris–HCl, pH 7.6, and 100 mM dithiothreitol (DTT). Lysates were incubated for 10 min at 95°C and then briefly sonicated.

For the comparison between 6537 and 6538 cells, we performed five biological replicates including label swap of heavy and light labeled cells, to eliminate potential effects of the labeling. In each replicate, equal protein amounts of heavy and light lysates were combined and the rest of the experimental procedure was performed for the combined lysate. For two replicates, lysates were separated by 6–12% gradient gel followed by gel staining with colloidal blue staining (Invitrogen). Each gel lane was cut into 10 fractions and proteins were trypsin-digested according to the in-gel digestion protocol [ref: PMID 17406544]. Three replicates were trypsin-digested in solution and peptides were separated using the off-gel fractionator [PMID:19003865]. In the analysis of patient samples, equal protein amounts of the nonlabeled lysates from the primary cells were combined heavy labeled lysate of 6537 cells that served as an internal standard for quantification [PMID: 21293456]. Lysates were digested using the FASP protocol [PMID: 19377485] followed by peptide separation into six fractions by strong anion exchange in a stageTip format [PMID: 19848406]. After each of the protocols, peptides were purified on C_18_ StageTips [PMID: 12585499] prior to MS analysis.

### LC-MS analysis

LC-MS analyses of the cell lines were performed on an Easy-nano-LC coupled to an LTQ Orbitrap XL mass spectrometer (Thermo Fisher Scientific). Peptides were separated on a C_18_ column (15 and 75 μm i.d. and 3 μm Reprosil resin), using a 100-min gradient of water/acetonitrile. Patient samples were analyzed on an EASY-nLC-1000 coupled to the Q-Exactive mass spectrometer (Thermo Fisher Scientific). Peptides were separated on a 50-cm C_18_ column (Dionex) using a 200-min gradient of water/acetonitrile.

### Data analysis

Raw MS data analysis was done in the MaxQuant environment [PMID: 19029910] ensuring 0.01 false discovery rate (FDR) on the peptide and protein levels.

For the analysis of NMD and UPR proteins in the patient samples and the deep proteomes of cell lines, we extracted the quantitative data of 71 selected proteins [NMD core proteins and UPR proteins which showed significantly different levels between 6537 and 6538 cell lines (Supplementary Table S3)]. Hierarchical clustering of proteins was performed on logarithmized ratios for the patient samples and logarithmized intensities for the cell line proteomes using Euclidean distances between averages. Prior to clustering, quantitative values were *z*-score-normalized on the protein and sample axes. Spearman's correlation between UPR and NMD proteins was determined on normalized values after averaging each group of proteins for each sample. Enrichment analysis was performed using Fisher's exact test with an FDR value of 0.02. Statistical analysis was done with the Perseus program in the MaxQuant environment [PMID: 21548781].

### Halide efflux assay (SPQ)

Cells were seeded onto Vectabond®-treated glass coverslips and grown to approximately 80% confluence. Immediately prior to study, cells were hypotonically loaded with halide-quenched dye (6-methoxy-*N*-(3-sulfopropyl) quinolinium, SPQ, 10 mM, Molecular Probes Inc., Eugene, OR) for 10 min and then placed in a quenching NaI-based buffer (King & Sorscher, 2000). CFTR robustly conducts iodide in addition to chloride, 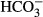, and other anions, allowing use of iodide quench as a measure of macroscopic channel activity. Cells were mounted in a specially designed perfusion chamber, and fluorescence was monitored using an inverted Nikon Diaphot microscope (Tokyo, Japan; excitation at 350 nm, emission at > 410 nm), an Easy Ratio Pro imaging system (PTI, Birmingham, NJ), and a CoolSNAP HQ_2_ camera (Photometrics, Tucson, AZ). Baseline fluorescence was initially studied in NaI buffer (above) followed by dequenching NaNO_3_ solution (King & Sorscher, 2000). CFTR agonists (20 μM forskolin, 50 μM genistein) were added to activate channel gating, after which NaI buffer was again perfused. Fluorescence was normalized for each cell versus baseline and increases shown as percent above basal (quenched) values. For each coverslip, > 15 individual cells were monitored. Average stimulated change in fluorescence from each coverslip was used for statistical analysis (*n* = 9–10 coverslips per condition; data recorded on five separate days). Data were expressed as mean ± s.e. and tested for significance using ANOVA. Results with *P *<* *0.05 were considered significant.

Since the SPQ assay is not well suited for multiple, timed drug additions, especially when some of the treatments (such as DTT) are borderline toxic and have their own potential for interrupting distinct targets in various pathways, we used TM in this set of experiments, which was less toxic to the cells.

### Sequence analysis of known NMD-triggering features

Deep intron is defined as any intron located more than 55 nucleotides downstream of a stop codon. A 3′UTR is considered long if its length is at least 1000 nt. uORF is an ORF upstream of the primary ORF (i.e., in the 5′UTR) that has an optimal Kozak signal (GCC[A/G]CCaugG[not U]). We tested all isoforms of ASNS, ATF3, ATF4, and CHOP taken from Ensembl (Flicek *et al*, 2013).

## The paper explained

### Problem

One-third of inherited diseases, caused by a defect in one gene, result from stop mutations (also defined as premature termination codons, PTCs). In recent years, an extensive effort has been made to develop therapeutic approaches for PTCs aimed to promote translational readthrough of the PTC and generate full-length functional proteins. Several readthrough approaches have been developed, including the use of aminoglycosides or ataluren. However, extended variability in the response to readthrough treatment is found among patients. Here, we aimed to reveal cellular pathways affecting this inter-patient variability.

### Results

We show that activation of the unfolded protein response (UPR), a homeostatic mechanism aimed to resolve protein-folding defects in the ER, governs the response to readthrough treatment by regulating the levels of transcripts carrying PTCs. Proteomic analyses showed substantial differences in UPR activation between patients carrying PTCs, correlating with their response. We further found a significant inverse correlation between the UPR and another homeostatic mechanism, the nonsense-mediated mRNA decay (NMD), suggesting a feedback loop between these pathways. We uncovered and characterized the mechanism underlying this NMD-UPR feedback loop. Importantly, this feedback loop enhances the response to readthrough treatment.

### Impact

These results provide new insights into the cellular mechanisms and the interplay between them in regulating readthrough and may enable the development of novel drugs aimed to improve the response to readthrough therapies for many human genetic diseases caused by PTCs.
