# Supplementary material for: The unfolded protein response affects readthrough of premature termination codons
Source: EMBO Mol Med. 2014 Apr 4;6(5):685–701. doi: 10.1002/emmm.201303347 (PMC4023889; doi:10.1002/emmm.201303347)
Supplement: Supplementary file 1 [file emmm0006-0685-sd1.pdf]

## Figure S1: The effect of UPR activation on the XLF function following G418 treatment

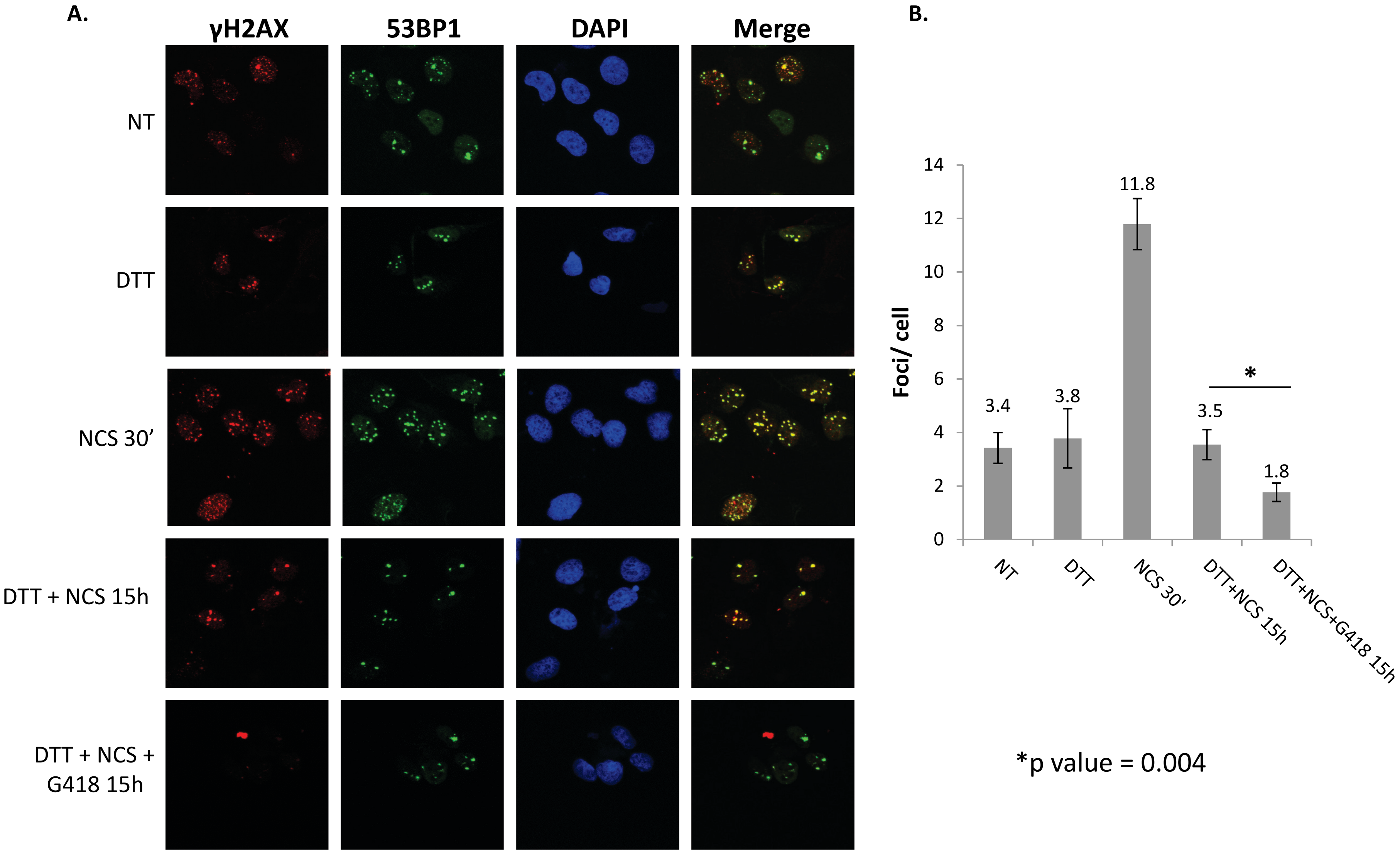

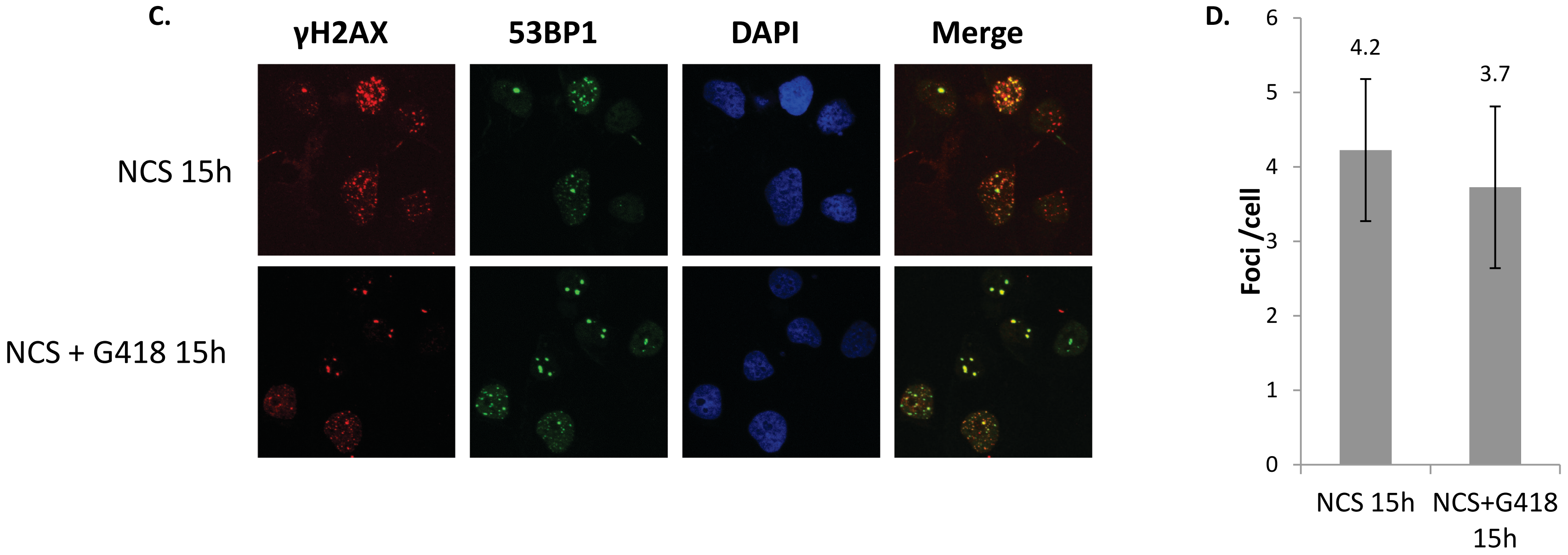


**Figure S1. The effect of UPR activation on XLF function following G418 treatment.**

P133 cells were grown and treated as described in Figure 3A. **(A, C)** Cells were fixed and immune-stained anti- γH2AX and anti-53BP1 antibodies. **(B, D)** Quantification of the number of colocalized γH2AX and 53BP1 foci.
