# Supplementary material for: The unfolded protein response affects readthrough of premature termination codons
Source: EMBO Mol Med. 2014 Apr 4;6(5):685–701. doi: 10.1002/emmm.201303347 (PMC4023889; doi:10.1002/emmm.201303347)
Supplement: Supplementary file 2 [file emmm0006-0685-sd2.pdf]

Figure S2: The effect of G418, TM, DTT and NMD inhibition on transcripts levels

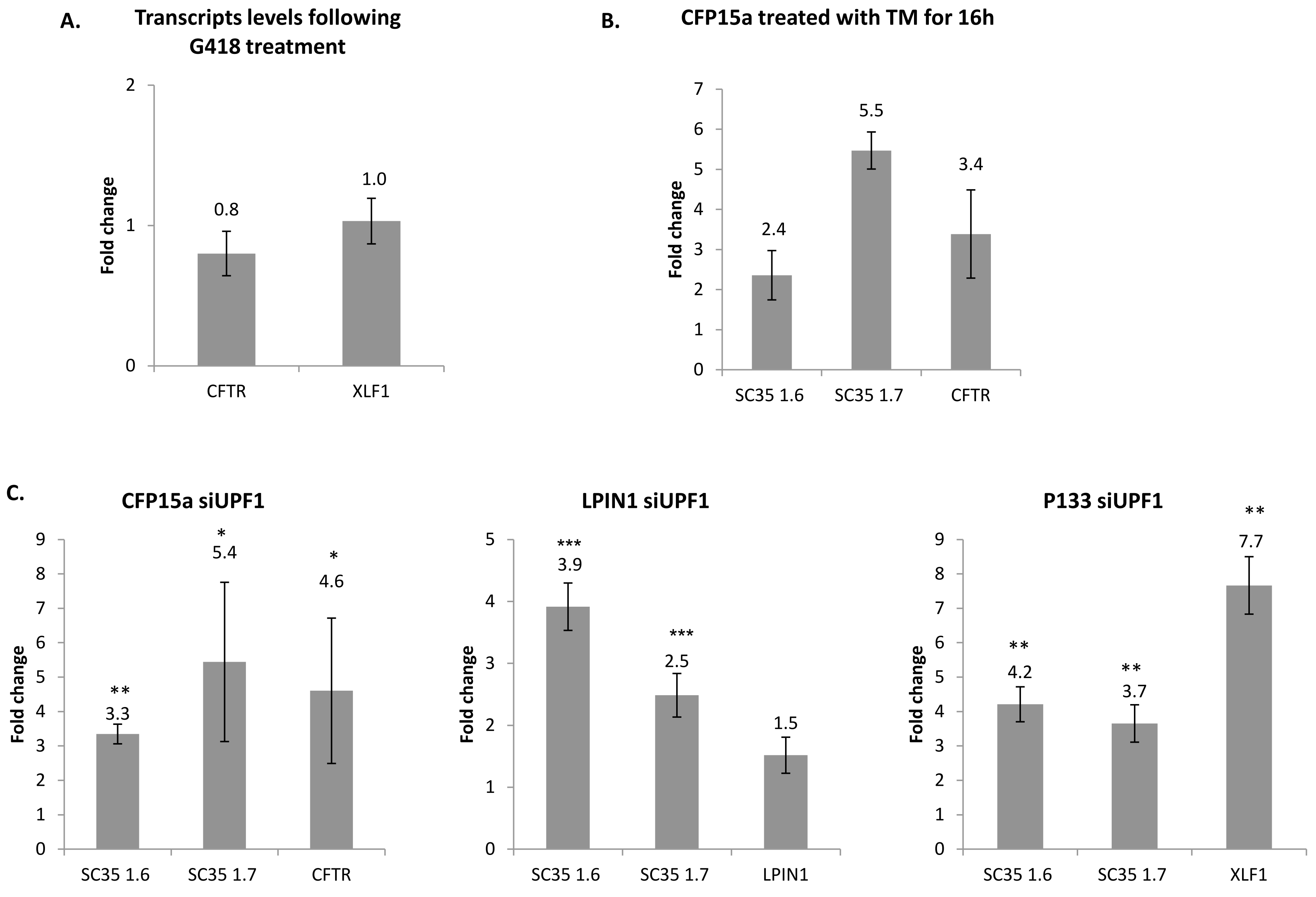

D.

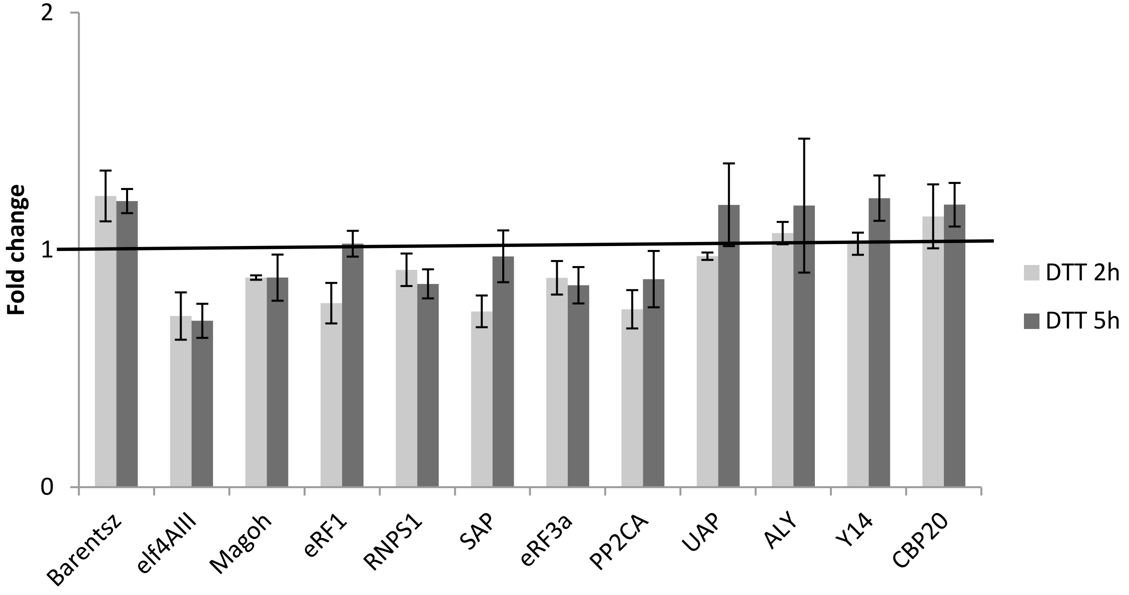

Figure S2. The effect of G418, TM, DTT and NMD inhibition on transcripts levels. (A) CFP15a and P133 cells were treated with G418 (250 µg/ml) for 48 and 24 hours respectively. **(B)** CFP15a cells were treated with TM for 16 hours. **(C)** CFP15a, LPIN1 and P133 cells were transfected with siRNA against hUPF1 or non-specific control siRNA (scr) for 72h (CFP15a, P133) or 96h (LPIN1). The values shown are the average fold change (mean±SEM) from at least three independent experiments relative to non-treated cells. Values were normalized against transcripts of RNA polymerase II gene. Statistical analysis was performed using Student's t test (1 tail, paired). $*p<0.05$, $**p<0.01$, $***p<0.001$. **(D)** HeLa cells were treated with DTT for 2 or 5 hours. The levels of the indicated transcripts were measured by RT-qPCR. The values shown are the average fold change (mean±SEM) from at least three independent experiments relative to non-treated cells. Values were normalized against transcripts of RNA polymerase II gene.
