# Supplementary material for: The unfolded protein response affects readthrough of premature termination codons
Source: EMBO Mol Med. 2014 Apr 4;6(5):685–701. doi: 10.1002/emmm.201303347 (PMC4023889; doi:10.1002/emmm.201303347)
Supplement: Supplementary file 3 [file emmm0006-0685-sd3.pdf]

Figure S3: Activation of UPR inhibits the NMD mechanism

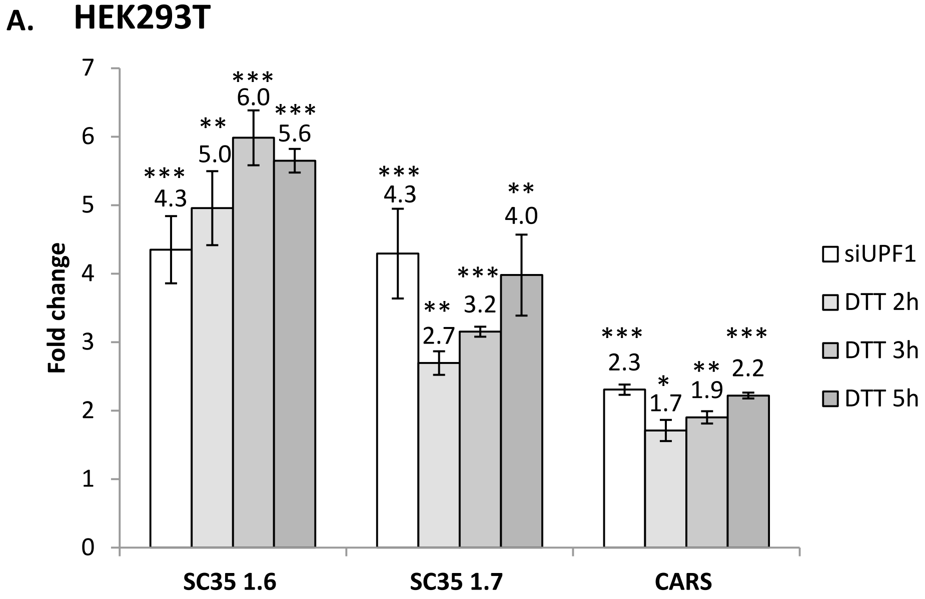

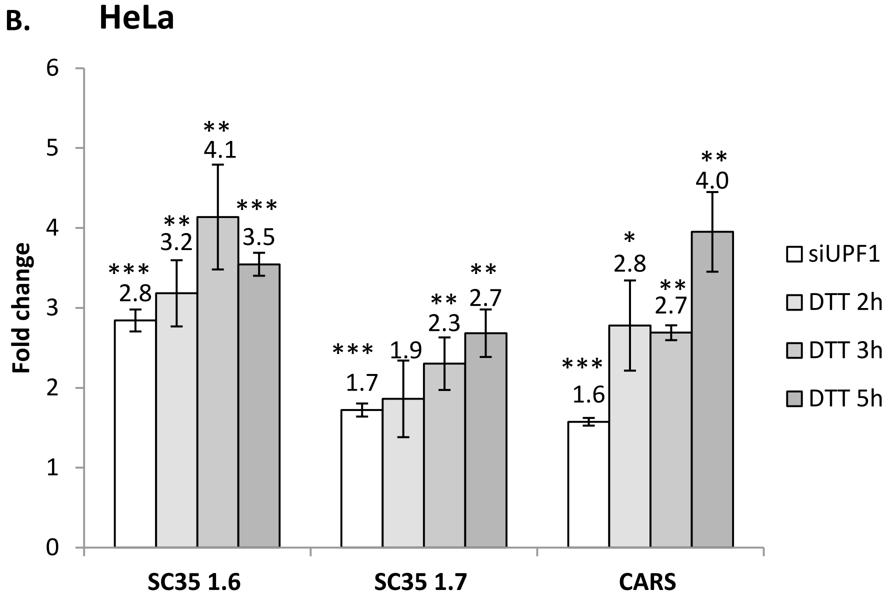

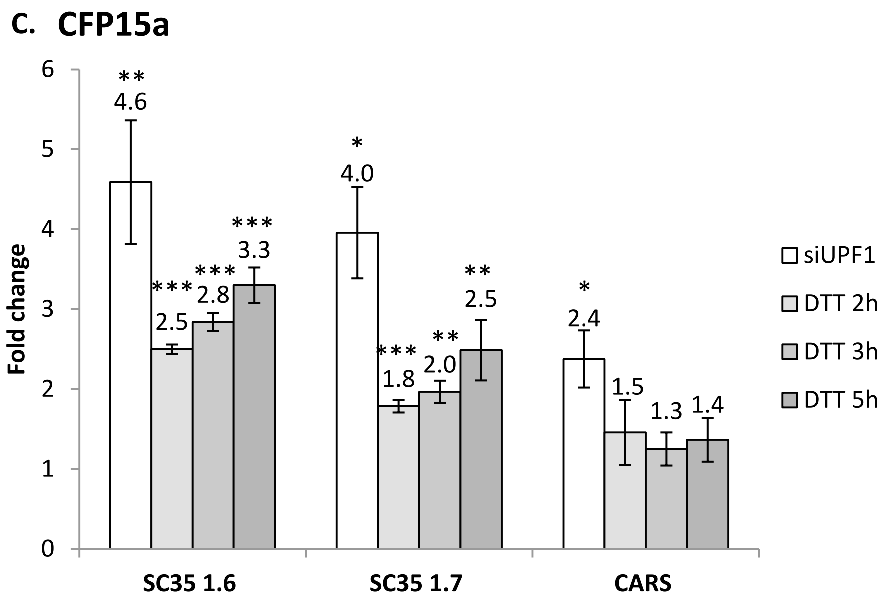

Figure S3. Activation of UPR inhibits the NMD mechanism. **(A)** HEK293T, **(B)** HeLa and **(C)** CFP15a cells were treated with DTT (10mM) for 2h, 3h and 5h or transfected with siRNA against hUPF1 or non-specific control siRNA (scr) for 48h (HeLa) or 72h (HEK293 and CFP15a). The levels of SC35 1.6, SC35 1.7 and CARS transcripts were measured by RT-qPCR. The values shown are the average fold change (mean±SEM) from at least three independent experiments relative to non-treated cells. Values were normalized against transcripts of RNA polymerase II gene. Statistical analysis was performed using Student's t test (1 tail, paired). *p<0.05, **p<0.01, ***p<0.001.
