# Supplementary material for: The unfolded protein response affects readthrough of premature termination codons
Source: EMBO Mol Med. 2014 Apr 4;6(5):685–701. doi: 10.1002/emmm.201303347 (PMC4023889; doi:10.1002/emmm.201303347)
Supplement: Supplementary file 4 [file emmm0006-0685-sd4.pdf]

Figure S4: NMD inhibition activates the IRE1-splised XBP1 UPR branch

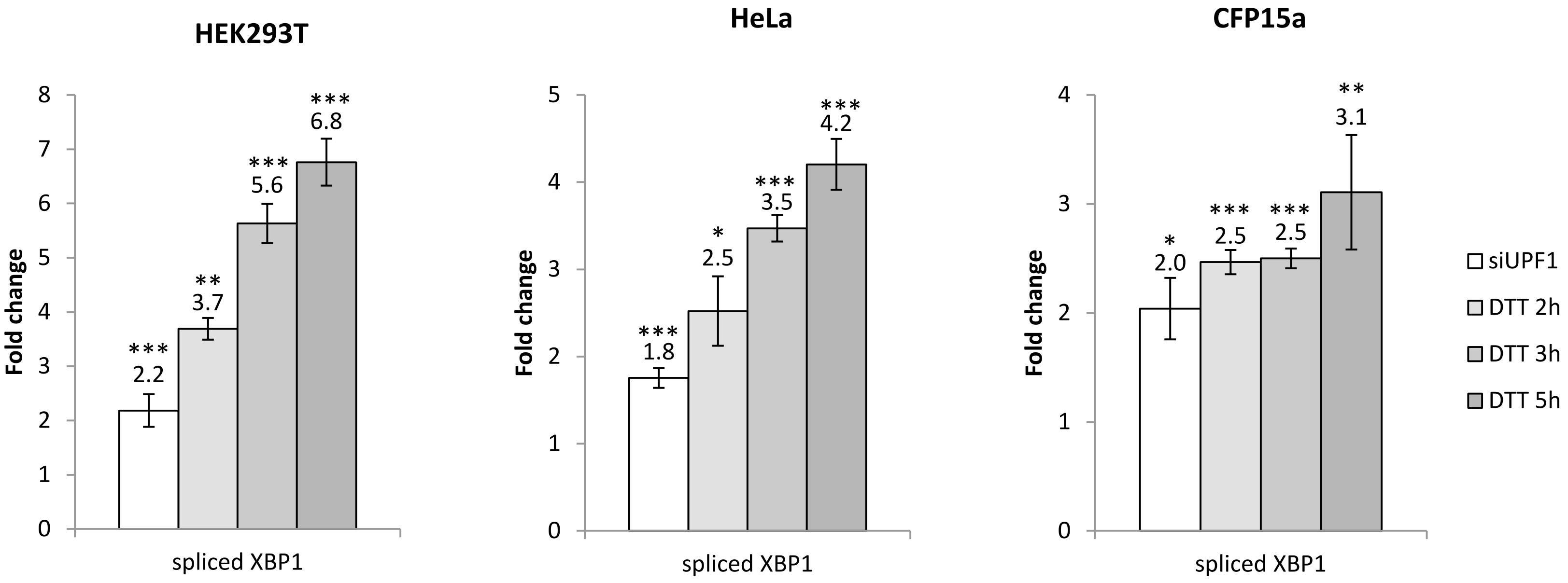

Figure S4. NMD inhibition activates the IRE1-spliced XBP1 UPR branch. HEK293T, HeLa and CFP15a cells were treated with DTT (10mM) for 2h, 3h and 5h or transfected with siRNA against hUPF1. The level of spliced XBP1 transcripts was measured by RT-qPCR. The values shown are the average fold change (mean±SEM) from at least three independent experiments relative to non-treated cells or cells transfected with non-specific control siRNA. Values were normalized against RNA Polymerase II transcripts (HEK293T and HeLa) or GAPDH (CFP15a). The values shown are the average fold change (mean±SEM) from at least three independent experiments relative to non-treated cells. Values were normalized against transcripts of RNA polymerase II gene. Statistical analysis was performed using Student's t test (1 tail, paired). *p<0.05, **p<0.01, ***p<0.001.
