# Supplementary material for: The unfolded protein response affects readthrough of premature termination codons
Source: EMBO Mol Med. 2014 Apr 4;6(5):685–701. doi: 10.1002/emmm.201303347 (PMC4023889; doi:10.1002/emmm.201303347)
Supplement: Supplementary file 5 [file emmm0006-0685-sd5.pdf]

Figure S5: Quantification of peIF2α levels following NMD inhibition and UPR activation

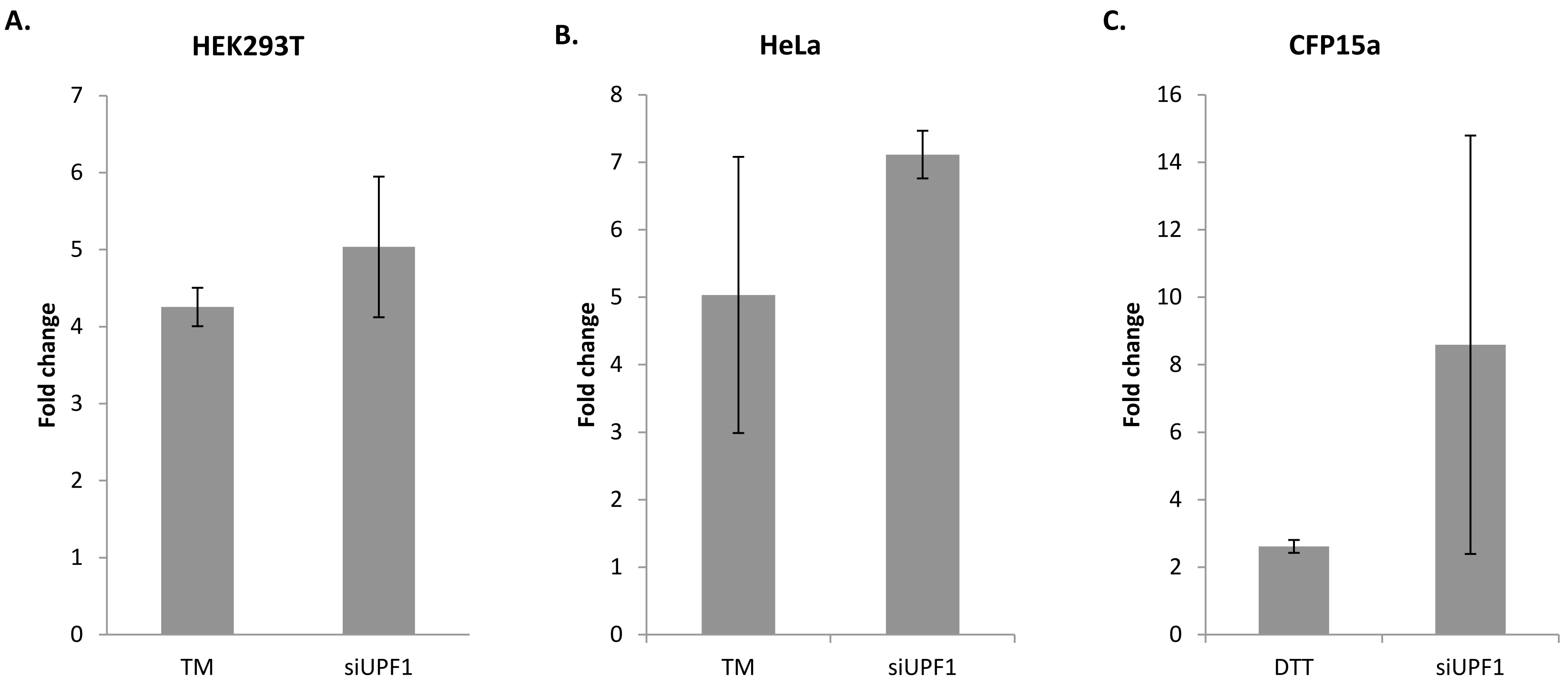

Figure S5. Quantification of peIF2α levels following NMD inhibition and UPR activation. Quantification of the western blots presented in Figure 5A and C. The levels of peIF2α were normalized to Tubulin. Quantification represents the average of at least two experiments.
