# Supplementary material for: The unfolded protein response affects readthrough of premature termination codons
Source: EMBO Mol Med. 2014 Apr 4;6(5):685–701. doi: 10.1002/emmm.201303347 (PMC4023889; doi:10.1002/emmm.201303347)
Supplement: Supplementary file 6 [file emmm0006-0685-sd6.pdf]

Figure S6: Quantification of CFTR levels following UPR activation and readthrough treatment

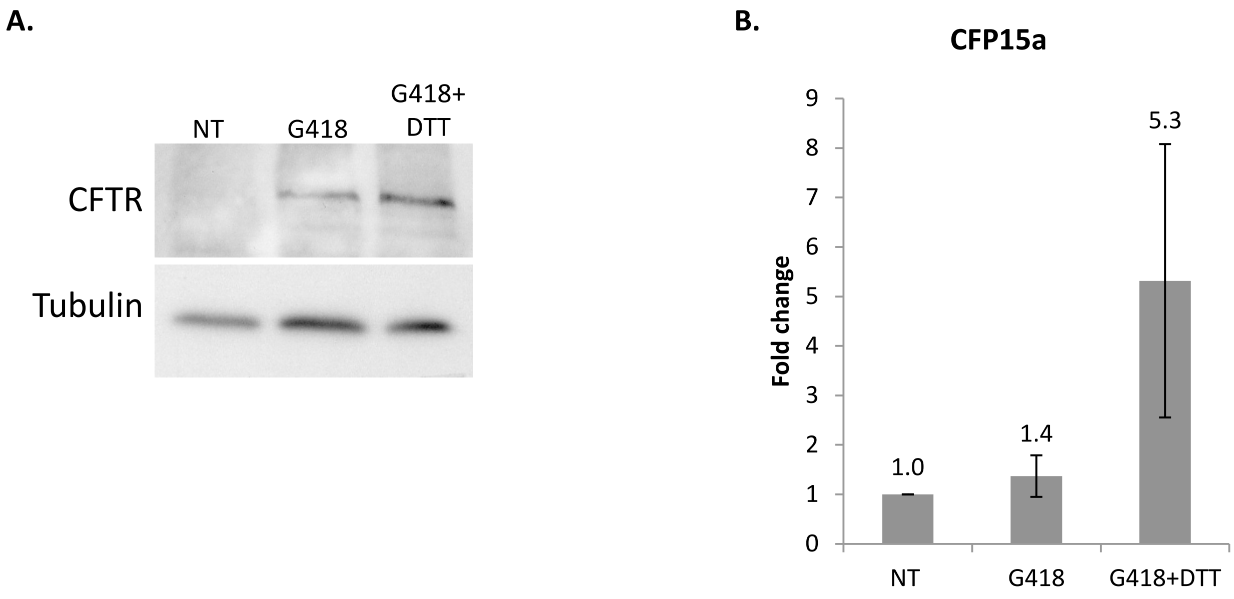

Figure S6. Quantification of CFTR levels following UPR activation and readthrough treatment. (A) Representative Western blot of CFTR protein levels in CFP15a cells following G418 treatment for 48 hours alone or combined with DTT treatment for 3 hours. **(B)** Quantification of CFTR levels normalized to Tubulin. Quantification represents the average of two experiments.
