# Supplementary material for: The unfolded protein response affects readthrough of premature termination codons
Source: EMBO Mol Med. 2014 Apr 4;6(5):685–701. doi: 10.1002/emmm.201303347 (PMC4023889; doi:10.1002/emmm.201303347)
Supplement: Supplementary file 7 [file emmm0006-0685-sd7.pdf]

Table S1: Examples of SILAC results - UPR proteins that are significantly different according to the SILAC-based proteomic analysis.

UPR function	Gene name	Ratio (6537/6538)	Significant
**Chaperone**	PDI 4	0.48043	8.18E-07
	BIP (GRP78)	0.57458	0.0001312
	calreticulin	0.61207	0.0006794
	Hsp40	0.61127	0.00523
	Hsp90	0.54653	0.0000359
	Hsp70	0.53123	0.0008272
	DnaJ	0.56167	0.005583
**Lipid biosynthesis**	Fatty acid desaturase 2	0.623	0.027964
	LACS 4	0.44778	0.0000139
	APOL 2	0.443	0.0000259
**ERAD**	UBE2J1 (E2)	0.38296	1.75E-07
