# Supplementary material for: The unfolded protein response affects readthrough of premature termination codons
Source: EMBO Mol Med. 2014 Apr 4;6(5):685–701. doi: 10.1002/emmm.201303347 (PMC4023889; doi:10.1002/emmm.201303347)
Supplement: Supplementary file 8 [file emmm0006-0685-sd8.pdf]

Table S2: Enrichment of Gene Ontology annotations

Category value	Enrichment factor	P value	Benj. Hoch. FDR
epidermis development	10.277	5.4102E-08	0.00019174
intermediate filament	8.5639	3.7357E-09	2.4656E-06
tissue development	6.978	1.8996E-07	0.00033661
cellular di-, tri-valent inorganic cation homeostasis	6.6608	0.000047093	0.012363
cell surface	6.3944	0.000062607	0.0051651
di-, tri-valent inorganic cation homeostasis	6.3944	0.000062607	0.014792
response to virus	4.8937	0.000055231	0.013499
response to other organism	4.8309	9.5696E-06	0.0042393
extracellular region	4.1814	2.4112E-10	3.1828E-07
response to biotic stimulus	3.7234	6.9996E-06	0.0038164
multi-organism process	3.6539	0.000067783	0.015498
calcium ion binding	3.3511	1.5918E-08	0.000062082
response to external stimulus	3.3218	0.000015127	0.0056433
defense response	3.2624	0.000018929	0.0058334
lipid metabolic process	2.708	3.1098E-07	0.00031489
anatomical structure development	2.6443	0.000016783	0.0056646
immune system process	2.61	8.7884E-06	0.0041528
cellular lipid metabolic process	2.5258	0.000015047	0.0059253
endoplasmic reticulum part	2.5027	0.000017454	0.0016456
endoplasmic reticulum membrane	2.4324	0.00025727	0.01415
regulation of apoptosis	2.4094	0.00004612	0.012573
regulation of developmental process	2.4083	5.0686E-06	0.0029939
regulation of programmed cell death	2.3875	0.000052874	0.013385
endoplasmic reticulum	2.3405	2.1068E-06	0.00034762
cytoskeletal part	2.0196	0.00025359	0.014554
response to stimulus	1.9525	2.3795E-07	0.00033732
developmental process	1.9404	8.1956E-07	0.0005809
cell junction	3.825	0.00041384	0.01951
plasma membrane part	2.6629	9.4594E-06	0.0009605

Table S2. Fisher Exact Test examined enrichment of Gene Ontology annotations in the set of proteins that are more highly expressed in patients 6538 or 6537. Benjamini Hochberg FDR was set to 0.002.
