# Supplementary material for: The unfolded protein response affects readthrough of premature termination codons
Source: EMBO Mol Med. 2014 Apr 4;6(5):685–701. doi: 10.1002/emmm.201303347 (PMC4023889; doi:10.1002/emmm.201303347)
Supplement: Supplementary file 9 [file emmm0006-0685-sd9.pdf]

Table S3: NMD and UPR factors

Gene Names	
**NMD factors**	**UPR factors**
Upf1, NORF1, RENT1	SDF2L1;UNQ1941/PRO4424
Upf2, RENT2, smg-3	TXNDC5;TLP46;UNQ364/PRO700
Upf3b, UPF3X, MRXS14, RENT3B	NT5C3;P5N1;UMPH1;HSPC233
SMG1, ATX, LIP	UBE2J1;NCUBE1;CGI-76;HSPC153;HSPC205
SMG7, EST1C, SGA56M	RRBP1;KIAA1398
Magoh	ACSL4;ACS4;FACL4;LACS4
RNPS1	ACSL3;ACS3;FACL3;LACS3
PYM, WIBG	PDIA4;ERP70;ERP72
U2AF2, UAP56, U2AF65	CYB5A;CYB5
TAP, p15, NXF1, MEX67	RTN4;KIAA0886;NOGO;My043;SP1507;hCG_1784307;NOGOC
SRm160, SRRM1, 160-KD, POP101	DHCR24;KIAA0018
Pinin, PNN, DRS, DRSP, SDK3, memA	CKAP4
Acinus, ACIN1, CAN, fSAP152	SCD
SAP18, SAP18P, 2HOR0202	HSPA1A;HSPA1;HSPA1B;HSP70-1;DAQB-147D11.2-002
PP2CA, PPP2CA, RP-C, PP2Ac, PP2Calpha	HSP90B1;TRA1
P29, SYF2, CBPIN, NTC31, fSAP29	PTPN1;PTP1B
CBP20, NCBP2, CBC2, NIP1, CBP20, PIG55	CRELD2;UNQ185/PRO211
CBP80, NCBP1, NCBP, Sto1	DNAJC3;P58IPK;PRKRI
eRF1, ETF1, ERF, RF1, ERF1, TB3-1, D5S1995, SUP45L1	CTSZ
eRF3a, GSPT1, GST1, ETF3A, eRF3a, 551G9.2	HSPA5;GRP78
	FADS2
	DHCR7;D7SR
	CASP7;MCH3;RP11-211N11.6-004
	ACSL5;ACS5;FACL5;UNQ633/PRO1250
	DNAJB11;EDJ;ERJ3;UNQ537/PRO1080
	OAS2
	CALR;CRTC
	ALDH3A2;ALDH10;FALDH
	HMOX1;HO;HO1
	MTDH;AEG1;LYRIC
	MGST1;GST12;MGST
	LPGAT1;FAM34A;KIAA0205
	ANK1;ANK
	PLD1
	RAB38;RAB32
	HSP90AB1;RP1-302G2.1-007;RP1-302G2.1-002;RP1-302G2.1-001
