# Supplementary material for: The unfolded protein response affects readthrough of premature termination codons
Source: EMBO Mol Med. 2014 Apr 4;6(5):685–701. doi: 10.1002/emmm.201303347 (PMC4023889; doi:10.1002/emmm.201303347)
Supplement: Supplementary file 10 [file emmm0006-0685-sd10.pdf]

Table S4: Sequences of all primers used in RT-qPCR

POLR2A: F: 5’ TGCGCA CCATCAAGAGAGTC 3’
R: 5’ CTCCGTCACAGACATTCGCTT 3’.
GAPDH: F: 5’ TGAGCTTGACAAAGTGGTCG 3’
R: 5’ GGCTCTCCAGAACATCATCC 3’
Spliced XBP1: F: 5’ CTGCTGAGTCCGCAGCAGGTG 3’
R: 5’ GGTCCAAGTTGTCCAGAATGC 3’
SC35 1.6: F: 5’ CCTCTTAAGAAAATGATGTAT 3’
R: 5’ CTGCTACACAACTGCGCC 3’
SC35 1.7: F: 5’ GTGGGCGTGTATTGGAGCAGA 3’
R: 5’ CTGCTACACAACTGCGCC 3’
CARS: F: 5’ TGTGAATGTTCTCCCTCCAGA 3’
R: 5’ GGCACATACTCACTAACCCGG 3’
ASNS: F: 5’ TGCAATTGGCATGGAAGACA 3’
R: 5’ GCCACCTTTCTAGCAGCCAG3’
ATF3: F: 5’ AAGGATTTTCAGCACCTT 3’
R: 5’ GATGGCAGAAGCACTCAC 3’
ATF4: F: 5’ TCTCCAGCGACAAGGCTA3’
R: 5’ CAATCTGTCCCGGAGAAG 3’
CHOP: F: 5’ CAGAGCTGGAACCTGAGG 3’
R: 5’ CTGCAGTTGGATCAGTCT 3’
UPF2: F: 5’ GGATGTTGGGATCCACGT 3’
R: 5’ CGCCTCTGATTAAATTTA 3’
Upf3a: F: 5’ CAGCACACGACTACTTCGAGTTCT 3’
R: 5’ CCTAAAATTAATCTATGCTCTTGAGTAGAGA 3’
Upf3b : F: 5’ GAAGATAAGCAGGATCGCAACAA 3’
R: 5’ GAAGCTGCTCCTTGGTCAAAGT 3’
SMG1 : F: 5’ CAGAGTCCACGCTGACATACAGA 3’
R: 5’ CCCAACGACTTCCGACCATA 3’
SMG5 : F: 5’ GTTGGCATCTTCGTCAGCATT 3’
R: 5’ AGCCTGTTCCGACGAGCTT 3’
SMG6: F: 5’ CAGCACATTGCTCCCAAGAA 3’
R: 5’ GCGCATATAGTAATAGACAGCGTCAA 3’
SMG7: F: 5’ GGTTGATTTCTCTTCTGAATAGTTTCC 3’
R: 5’ TCCTTGTAATTCAAACTCCTCTGGA 3’
CFTR: F: 5’ AATGCTGGAATGCCAACAATT 3’
R: 5’ GGCTCCTCTCGTTCAGCAGT3’
LPIN1: F: 5’ TGGAATGGAGGTATATCATTAGGAA3’
R: 5’ CGAGATGAGCTCGGATGAG3’
XLF1: F: 5’ TGGCATGAACAGGTGGACACT 3’
R: 5’ GCCGCTTGTTCAGCTCCTT 3’
