# Supplementary material for: The unfolded protein response affects readthrough of premature termination codons
Source: EMBO Mol Med. 2014 Apr 4;6(5):685–701. doi: 10.1002/emmm.201303347 (PMC4023889; doi:10.1002/emmm.201303347)
Supplement: Supplementary file 11 [file emmm0006-0685-sd11.pdf]

Table S5: Actual P values for each experiment

Figure no.	Cell line	Gene	n	p. value
4	CFP15a	SC35 1.6 2h	4	1.87E-05
		SC35 1.6 3h	4	7.70E-04
		SC35 1.6 5h	5	3.04E-05
		SC35 1.7 2h	4	4.84E-04
		SC35 1.7 3h	4	5.71E-03
		SC35 1.7 5h	5	1.54E-03
		CFTR 2h	4	1.75E-02
		CFTR 3h	4	7.14E-04
		CFTR 5h	5	8.16E-03
	LPIN1	SC35 1.6 2h	4	2.45E-03
		SC35 1.6 3h	4	3.92E-03
		SC35 1.6 5h	4	4.19E-04
		SC35 1.7 2h	4	4.39E-04
		SC35 1.7 3h	4	1.59E-03
		SC35 1.7 5h	4	1.04E-04
		LPIN 1 2h	4	2.16E-03
		LPIN 1 3h	4	2.19E-02
		LPIN 1 5h	4	5.12E-05
	P133	SC35 1.6 2h	3	9.13E-04
		SC35 1.6 3h	3	5.21E-05
		SC35 1.6 5h	3	1.50E-02
		SC35 1.7 2h	3	4.37E-02
		SC35 1.7 3h	3	2.24E-03
		SC35 1.7 5h	3	5.72E-02
		XLF1 3h	3	5.75E-03
		XLF1 5h	3	7.57E-04
5	HEK293	SC35 1.6	6	1.57E-05
		SC35 1.7	6	3.23E-05
		CARS	6	4.11E-05
	HeLa	SC35 1.6	7	3.93E-07
		SC35 1.7	6	4.32E-05
		CARS	5	6.36E-05
	CFP15a	SC35 1.6	4	2.71E-03
		SC35 1.7	4	2.67E-02
		CARS	4	1.36E-02
6	HEK293	ATF3	3	7.81E-03
		ATF4	3	2.35E-03
		CHOP	3	4.07E-02
		ASNS	3	1.21E-02
	HeLa	UPF2 siUPF1	3	2.70E-02
		UPF2 DTT 2h	3	4.90E-02
		UPF2 DTT 5h	3	1.05E-02
		UPF3a DTT 2h	3	4.93E-02
		UPF3b siUPF1	3	1.08E-02
		UPF3b DTT 2h	3	1.14E-02
		UPF3b DTT 5h	3	8.88E-04
		SMG1 siUPF1	3	9.12E-03
		SMG1 DTT 2h	3	1.79E-04
		SMG1 DTT 5h	3	1.80E-05
		SMG5 siUPF1	3	6.01E-03
		SMG5 DTT 2h	3	2.18E-02
		SMG5 DTT 5h	3	6.11E-04
		SMG6 DTT 2h	3	3.91E-03
		SMG6 DTT 5h	3	4.37E-04
		SMG7 DTT 2h	3	1.08E-02
		SMG7 DTT 5h	3	1.12E-02
	HEK293	spliced XBP1 (scr/siUPF1)	5	2.97E-02
		SC35 1.6 siUPF1/scr 2h	6	9.18E-05
		SC35 1.6 siUPF1/scr 2h	6	3.28E-04
		SC35 1.6 siUPF1/scr 3h	5	1.44E-03
		SC35 1.6 siUPF1/scr 3h	5	6.38E-04
		CARS siUPF1/scr 2h	6	2.96E-02
		CARS siUPF1/scr 2h	6	2.68E-02
		CARS siUPF1/scr 3h	5	2.92E-02
		CARS siUPF1/scr 3h	5	1.73E-02

Figure no.	Cell line	Gene	n	p. value
6	HEK293	SMG1 siUPF1/scr 2h	6	1.27E-02
		SMG1 siUPF1/scr 2h	6	2.06E-03
		SMG1 siUPF1/scr 3h	4	5.13E-03
		SMG1 siUPF1/scr 3h	4	1.34E-02
		ATF3 siUPF1/scr 2h	5	5.61E-03
		ATF3 siUPF1/scr 2h	5	2.76E-03
		ATF3 siUPF1/scr 3h	5	4.98E-04
		ATF3 siUPF1/scr 3h	5	7.92E-03
		SC35 1.6 siPERK/scr 3h	3	1.56E-02
		CARS siPERK/scr 3h	3	3.05E-02
		SMG1 siPERK/scr 3h	3	2.66E-03
		spliced XBP1 (scr/siPERK)	3	5.00E-02
	6538/6537	spliced XBP1	3	1.88E-02
S2	CFP15a	SC35 1.6	3	2.71E-03
		SC35 1.7	3	2.67E-02
		CFTR	3	4.98E-02
	LPIN1	SC35 1.6	3	1.94E-05
		SC35 1.7	3	9.54E-04
	P133	SC35 1.6	3	3.26E-03
		SC35 1.7	3	6.71E-03
		XLF11	3	1.50E-03
S3	HEK293	SC35 1.6 siUPF1	6	1.57E-05
		SC35 1.6 2h	3	3.72E-03
		SC35 1.6 3h	3	7.46E-04
		SC35 1.6 5h	3	1.61E-04
		SC35 1.7 siUPF1	7	3.23E-05
		SC35 1.7 2h	3	4.14E-03
		SC35 1.7 3h	3	2.08E-04
		SC35 1.7 5h	3	5.41E-03
		CARS siUPF1	8	4.11E-05
		CARS 2h	3	1.32E-02
		CARS 3h	3	2.74E-03
		CARS 5h	3	2.90E-04
	HeLa	SC35 1.6 siUPF1	7	3.93E-07
		SC35 1.6 2h	3	7.30E-03
		SC35 1.6 3h	3	2.23E-03
		SC35 1.6 5h	3	3.33E-05
		SC35 1.7 siUPF1	6	4.32E-05
		SC35 1.7 3h	3	6.47E-03
		SC35 1.7 5h	3	1.39E-03
		CARS siUPF1	5	6.36E-05
		CARS 2h	3	1.84E-02
		CARS 3h	3	9.50E-03
		CARS 5h	3	1.02E-03
	CFP15a	SC35 1.6 siUPF1	4	2.71E-03
		SC35 1.6 2h	4	1.87E-05
		SC35 1.6 3h	3	7.70E-04
		SC35 1.6 5h	5	3.04E-05
		SC35 1.7 siUPF1	4	2.67E-02
		SC35 1.7 2h	4	4.84E-04
		SC35 1.7 3h	3	5.71E-03
		SC35 1.7 5h	5	1.54E-03
		CARS siUPF1	3	1.36E-02
S4	HEK293	spliced XBP1 siUPF1	8	1.39E-05
		spliced XBP1 2h	3	4.10E-03
		spliced XBP1 3h	3	6.78E-04
		spliced XBP1 5h	3	5.95E-04
	HeLa	spliced XBP1 siUPF1	7	4.96E-05
		spliced XBP1 2h	3	1.56E-02
		spliced XBP1 3h	4	4.39E-05
		spliced XBP1 5h	4	1.42E-04
	CFP15a	spliced XBP1 siUPF1	4	2.91E-03
		spliced XBP1 2h	4	1.47E-04
		spliced XBP1 3h	3	8.11E-04
		spliced XBP1 5h	5	1.16E-03

(**n**) = number of independed experiments
