# Supplementary material for: The unfolded protein response affects readthrough of premature termination codons
Source: EMBO Mol Med. 2014 Apr 4;6(5):685–701. doi: 10.1002/emmm.201303347 (PMC4023889; doi:10.1002/emmm.201303347)
Supplement: Supplementary file 12 [file emmm0006-0685-sd12.pdf]

EMBO Molecular Medicine

The Unfolded Protein Response affects readthrough of Premature Termination Codons

Yifat S Oren, Michelle L McClure, Steven M Rowe, Eric J Sorscher, Assaf C Bester , Miriam Manor, Eitan Kerem, Joseph Rivlin, Fouad Zahdeh, Matthias Mann, Tamar Geiger, Batsheva Kerem

Corresponding author: Batsheva Kerem, The Hebrew University -Member

Review timeline:

Submission date:	04 August 2013
Editorial Decision:	30 August 2013
Revision received:	04 February 2014
Editorial Decision:	18 February 2014
Revision received:	20 February 2014
Editorial Decision:	21 February 2014
Revision received:	03 March 2014
Accepted:	06 March 2014

Transaction Report:

(Note: With the exception of the correction of typographical or spelling errors that could be a source of ambiguity, letters and reports are not edited. The original formatting of letters and referee reports may not be reflected in this compilation.)

Editor: Roberto Buccione

1st Editorial Decision 30 August 2013

Thank you for the submission of your manuscript to EMBO Molecular Medicine.

We have now heard back from the three Reviewers whom we asked to evaluate your manuscript.

You will see that all three are generally supportive of you work but raise significant issues that question the conclusiveness of the results thus preventing us from considering publication at this time. I will not dwell into much detail, as the evaluations are detailed and self-explanatory. I would like, however, to highlight a few main points.

Reviewer 1 feels that further experimentation is required to support your contention of a feedback loop between nonsense mediated mRNA decay (NMD) and the UPR and suggests some approaches to that effect. Indeed, I should add that this concern appears to be a leitmotif among the Reviewers. Reviewer 1 notes additional issues that require your action.

Reviewer 2 is also concerned about the NMD/UPR feedback loop and actually suggests that it is not required for the manuscript, provided that the translational aspects of your work are further developed. S/he notes, however, that should you prefer to maintain the point on the NMD/UPR relationship, further mechanistic analysis is required. I would encourage you to maintain this part, provided you develop it further as the two Reviewers suggest. This Reviewer also lists other well-taken and critical points that require your intervention.

Reviewer 3 also notes that additional data is required to support the conclusions. For instance, s/he feels that direct demonstration is required to maintain that readthrough by G418 is improved when UPR is activated. This Reviewer, similarly to Reviewer 2, requires an explanation for the alternated use of tunicamycin and DTT. S/he also provides a detailed list of other shortcomings for you to act upon to increase the overall quality of data and presentation.

Considered all the above, while publication of the paper cannot be considered at this stage, we would be prepared to consider a suitably revised submission, with the understanding that the Reviewers' concerns must be fully addressed with additional experimental data where appropriate and that acceptance of the manuscript will entail a second round of review.

Since the required revision in this case might require a significant amount of time, additional work and experimentation and might be technically challenging, I would therefore understand if you chose to rather seek publication elsewhere at this stage. Should you do so, we would welcome a message to this effect.

Please note that it is EMBO Molecular Medicine policy to allow a single round of revision only and that, therefore, acceptance or rejection of the manuscript will depend on the completeness of your responses included in the next, final version of the manuscript.

As you know, EMBO Molecular Medicine has a "scooping protection" policy, whereby similar findings that are published by others during review or revision are not a criterion for rejection. However, I do ask you to get in touch with us after three months if you have not completed your revision, to update us on the status. Please also contact us as soon as possible if similar work is published elsewhere.

***** Reviewer's comments *****

Referee #1 (Remarks):

This manuscript describes a study using a variety of cell lines that provides evidence for a relationship between NMD and the UPR response. This relationship is of clinical interest since it may contribute to the efficacy of translational read through therapeutics. Overall, I find the study to be potentially interesting. However many of the experiments that are performed with specific cell lines need additional controls or should be analyzed in more depth to provide convincing evidence for the conclusions that are drawn.

1. Fig. 1: The matched patient cell lines are potentially interesting - but the molecular underpinnings for the proteomic differences are not pursued. Are there potential candidate genes that regulate the UPR response that can be assessed for expression levels or other analyses that can be performed to use this nice cellular model to test aspects of their feedback loop model?
2. Fig. 2: The results with TM and G148 are interesting, but to really nail the connection between UPR and NMD, other treatments such as DTT and siRNA-mediated KDs should be performed in these cells.
3. Fig. 3: Statistics and error bars to indicate reproducibility must be added to support the conclusions drawn from these data.
4. Fig. 4: The authors should use Tunicamycin and UPR/NMD knockdowns to confirm the conclusions drawn from this experiment. In addition, please add some control transcripts to address whether this up-regulation is specific for NMD or a general effect in the cell.
5. Fig. 5 A & C: Please quantify the effects that are observed and add error bars/statistics to better document the 'substantial' increase in eIF2 phosphorylation that is concluded from these data. Also changing the * to p =0.05 rather than the conventional less than 0.05 (if I understand the legend correctly) is not appropriate.
6. Fig. 6: These data would be more effective if negative control transcripts were shown that are unrelated to NMD
7. Minor points
a. The grammar and word usage can use some polishing.
b. The legend to Fig. 4 and 5 states that qRT-PCR values were normalized against RNA Polymerase

II transcripts. Since every mRNA is an RNA Pol II transcript, please re-phrase

Referee #2 (Remarks):

During the last decades translational therapies appeared as a strategy of choice for diseases linked to a premature termination codon (PTC). This approach has been successful in many cases but show variable results from one patient to another. NMD, which is a surveillance pathway involved in the degradation of PTC-bearing mRNA is obviously an important mechanism to take into account to maximise protein expression by PTC readthrough. It has been previously shown in cancer cells that ER stress can inhibit NMD. In this study the authors found that UPR activation regulated the levels of mRNA carrying PTC by inhibiting NMD. They suggest that UPR pathway could be an important parameter to determine the success of readthrough strategies.
This is an interesting point, and they are right to highlight the potential interest of the UPR pathway for PTC treatments. However, the potential effect of the UPR on endogenous transcript seems very limited. Moreover the authors provide several claims, which make the manuscript less understandable for the reader. Indeed, they start from PTC diseases and their link with NMD to reach the identification of a potential feedback loop between NMD and UPR. Unfortunately this is only a speculative model, and there is no attempt to better characterize molecular mechanisms to demonstrate this model. In my opinion this part is out of the scope of the manuscript. I would suggest to the authors to focus more precisely onto the relation between PTC disease and UPR and how this UPR pathway can be of interest for translational suppression strategies. Regarding the title they chose they could easily cut the part about the feedback loop, which is not directly related to this main question unless they can provide a molecular explanation.

Specific comments

1- The introduction is logically focused on two genetic diseases (CFTR and DMD), however it would be also important for the reader to understand that this approach is not limited to genetic diseases and could be also applied to cancers. There are several studies that could be cited.
2- In the introduction (end of page 3) they indicate that the variable responses to the treatments using aminoglycosides would be linked to NMD. This is a simplistic view of this important issue. As they are certainly aware the response to the treatment strongly depends of the stop codon and its nucleotide context. This must be indicated in the introduction, to make clear that NMD is not the only explanation to explain this variability.
3- It is not clear why they use two different molecules to induce UPR. They start firs with Tunicamycin (Figure 2) and then they use DTT for the rest of the manuscript. This should be explained, or they must only use DTT to get comparable results.
4- Page 9 the authors claim that the level of -H2AX is increased following NCS treatment, in P133 cells and they reefer to the Figure 3.A, which is probably an error. Indeed the Figure 3.A shows a strong induction of -H2AX by DTT (compare first and second lanes of Figure 3.A). This induction of -H2AX is not discussed at all, and this is not clear for me how they can expect to see a reduction of the level of -H2AX if DTT alone provides such a strong stimulation. As the authors want to prove that G418+DTT result in a reduce level of -H2AX this is problematic. Moreover they should tone down their claim of a "considerably reduced level" as this is only a 2 fold change. There is no indication how many times this experiment has been done, and error bars on the figure 3.C would help to estimate the reproducibility as the tubulin level seems highly variable. Moreover the authors should provide the Fold change of -H2AX without NCS treatment to determine the significance of this 2 fold reduction.
5- In my opinion the feedback loop between NDM and UPR is out of the scope of this article. However if the authors decide to maintain it they have to remind that a correlation is not a demonstration, and they must be more cautious in their proposal of a direct feedback loop between NMD and UPR especially in absence of any molecular explanation.
6- Page 18. They indicate that the level of mRNA is a limiting factor for the response to the readthrough treatment. However, there are several recent reports indicating that low level of stop codon readthrough inhibits the NMD. This should be mentioned in the discussion and could be take into account in their model. In this way only the very first rounds of translation would be affected by the UPR activity.
7- Page 38. Figure 4, CARS in mentioned in the figure of the legend but is absent from the figure.

Why the experience using the siRNA UPF1 has not been done with CFP15a, LPIN1 and P133 cells? This has been done with other cell types in supplemental figure S1. This is a very good control experiment giving the upregulation of the mRNA when NMD is inactivated. This should be also shown to indicate the level of induction by NMD inhibition in the three cell lines.
8- it seems that Figure 5.D is indeed figure 6A, and the legend of figure 6.A corresponds to figure 6.B. The authors must check their figures and their corresponding legends.

Referee #3 (Remarks):

Manuscript from Oren et al. aims to demonstrate that a regulation exists of NMD on UPR and UPR on NMD. The interest of showing this regulation is to modulate the response to drugs activating PTC-readthrough by targeting the UPR mechanism. This work opens a new way to help the nonsense mutation therapeutic approach. Although, the demonstration that ER stress (which will lead to the activation of UPR) inhibits NMD is not new and was published 2 years ago by Wang and collaborators in a MCB paper. Here authors go further in the regulation by focusing on the UPR activation.
Several technical approaches have been used in this work and bring new knowledge and in particular the double regulation of NMD on UPR and UPR on NMD.
The topic of this manuscript is therefore interesting and some data need to be more supported by additional experiments or information:
_Indeed for figure 1, authors want to demonstrate that the difference of response to a treatment between the 2 sisters is due to 10% of the proteins that have a significant difference of expression between them. Authors should help the readers by also providing an analysis of the 90% of the remaining proteins that also have a significant difference between the 2 sisters. It would help understanding why authors focused on the UPR activation and not on another pathway.
_For the figures 2 and 3, authors treated cells carrying a nonsense mutation in the CFTR or XLF gene with either G418 or/and tunicamycin/DTT and analyze the functionality of CFTR or XLF. They show that the function of CFTR or XLF is improved when both G418 and tunicamycin/DTT are added to the cells compared to the functionality obtained when only one treatment is used. Since all the working hypothesis of this experiment is to demonstrate that readthrough of the nonsense mutation by G418 is improved when UPR is activated, the demonstration would be complete with an analysis of the level of the CFTR or XLF mRNAs and the CFTR or XLF proteins by RT-qPCR and Western-blot. Measure of mRNA level is absolutely necessary also because G418 has been reported to inhibit NMD too (Correa-Cerro et al., 2005) and the Western blot analysis will demonstrate the readthrough activity. The analysis of the function of CFTR or XLF is complementary but not the direct demonstration of the readthrough. By analyzing only the protein function, authors include several additional steps (maturation, transport and localization,...) that can interfere with the interpretation of the data for the readthrough activity.
_Authors should also provide an explanation why they used tunicamycin in the figure 1 and DTT in other figures?

_According to the figure 6A, authors conclude that NMD regulates key factors of the UPR pathway since after using siRNA against UPF1, these factors were up-regulated. Is there any molecular explanation for this regulation as it has been provided for SC35 for instance? It would be interesting to know whether these genes have a long 3'UTR or some specific splicing reactions introducing a premature termination codon? Without such explanation, it is difficult to know whether NMD regulates these gene expressions or if the up-regulation observed after UPF1 down-regulation is an indirect consequence?

Minor points:

_Some sentences are difficult to understand. For instance, Page 5, starting at line 4: the following sentence is not correct "These proteins are translocated in to the ER where they are translated,...". What authors mean by "proteins are translated"?
_page 11 line 5: "Although it is no wonder that translation inhibition causes a decrease in NMD, we speculated that this decrease would cause more truncated proteins to be translated.". How to explain that if the translation is inhibited, more proteins are going to be translated?

_The legend of the figure 6 is difficult to understand since the lettering does not correspond to the panels or the description of the legend.
_Last sentence before the discussion: "result ding" ->"resulting"
_Figure 3B: We cannot see which condition has G418 and which one does not have because the gel covers the description!
_Page 9, line 6: merge the 2 sets of references
_Page 22, line 7: "P133 cells were described previously described...", one "described" has to be removed
_Table S2 is not referenced in the manuscript

1st Revision - authors' response 04 February 2014

We have **fully addressed all the points raised by the three reviewers. We performed all the experimental suggestions as well as introduced all textual requirements.**

Importantly, we have accepted the editor's suggestion to retain the NMD-UPR feedback loop part. For this we have performed several crucial experiments that clearly support and strengthened the NMD-UPR loop (see the details below, in response to reviewer 1 point 1 and reviewer 2). We thank the reviewers for raising this point, since we feel that the new results clearly improve and further support our feedback loop model and highlight the functional role of the NMD-UPR feedback loop in modulating readthrough treatment.

We also addressed all the other points raised by the reviewers as detailed below.

Referee #1

We have performed all the experimental suggestions and added all the required controls to provide evidence for the conclusions as detailed below.

1. Figure 1: We first wish to thank the reviewer for this comment. Indeed, our additional analyses, as suggested by the reviewer, significantly contributed to our conclusion of NMD-UPR novel feedback loop model.

As suggested by the reviewer we have pursued the molecular basis underpinning the proteomic differences found between the matched patient cell lines by the following analyses:

1a. We have analysed the transcript level of spliced-XBP1 and found a higher level of this key UPR factor in the cell line derived from the responding sister (patient 6538) compared to the level in the non-responding sister (patient 6537). This result fully supports the differences found in the proteomic analyses (see new Figure 6I).

1b. We further identified higher transcript levels of known NMD substrates (Figure 6I) in the responding sister cells, supporting our feedback loop model in this matched patient cell lines.

1c. Both NMD and UPR factors are regulated by post translational modifications, including phosphorylations. Subsequent to the proteomic analyses, we have performed analyses of phosphorylated peptides using titanium dioxide phosphopeptide enrichment followed by mass spectrometric analysis. Overall, we identified thousands of phosphorylation sites, among those we found several interesting differences in the phosphorylation of important NMD and UPR factors (a Table summarizing these results can be found at the end of this letter). However, the role of the phosphorylation of these sites is not known yet. Although much is known about the mechanism of NMD, many mysteries still remain. Discovery of new posttranslational modifications of NMD factors and what role they play in regulating NMD activity will likely be another important avenue of research, which is beyond the scope of this manuscript. If the reviewer thinks that the Table should be added to the manuscript we will be glad to do so. We referred to this point in the discussion (please see page 21 last paragraph).

2. Figure 2: It is important for us to note that the SPQ assay is not well suited for multiple, timed drug additions, especially when some of the treatments (such as DTT) are borderline toxic and have their own potential for interrupting distinct targets in various pathways. Therefore, we used tunicamycin (TM) (and not DTT) to activate the UPR, which is a less destructive treatment for these

sensitive cells. Unfortunately, in the multiple experiments that we have performed the combined treatment of TM and siRNA mediated NMD inhibition in the SPQ functional system was toxic for these cells.

However, in order to nail the connection between UPR and NMD we performed the combined treatment of DTT and NMD inhibition in P133 cells (please see the new figure 9), which well shows an enhanced response of the cells to readthrough treatment, compared to the effect of each treatment alone (NMD inhibition or UPR activation) and sheds a new light on the functional role of the NMD-UPR feedback loop.

3. Figure 3: In order to indicate the reproducibility of the results presented in this figure we performed immunofluorescent analysis of gH2AX colocalized with 53BP1, for quantification of the DNA double strand break repair (please see new Figure S1 and in the text page 10 line 2 from the bottom). Importantly, as can be seen in the figure, UPR activation by DTT treatment together with readthrough by G418 resulted in significantly reduced levels of DNA double strand breaks concomitant with our results from Figure 3.

We added a schematic representation of the experiment's design to help the reader follow the details (Figure 3A).

4. As suggested by the reviewer we have now added experiments showing the effect of siRNA directed against hUPF1 in CFP15a, LIPIN1, P133 cells and of TM in CFP15a, on the level of the transcripts regulated by NMD (please see the new Figure S2). These results show that the effect of direct NMD inhibition is similar to the effect of UPR activation.

The reviewer also requested that control transcripts will be analysed to address whether NMD substrates upregulation is specific for NMD inhibition following UPR activation or whether it is a general effect in the cell. We thank the reviewer for this comment. We originally performed these controls but did not include them in the first version of the manuscript. We have now included the results in the manuscript, in Figure S2D and in the Result section page 12, seven lines from the bottom of the page). The results show that DTT treatment at various times did not lead to any change in the level of a large battery of different "control" transcripts (more than 10), indicating that the increased level of NMD substrates results from NMD inhibition due to UPR activation and is not a general effect of DTT treatment in the cells.

5. Figure 5 A and C: as requested, we have added quantification of the observed effect and error bars, please see new Figure S5. We referred to this in the text (page 14 line 11 and 13 from top).

We would like to emphasize that in all experiments the asterisk refers to a p- value<0.05. While in the experiment presented in Figure 6H the p-value was 0.05. Therefore, as suggested by the reviewer we omitted the asterisk from this figure.

6. Please see response to Figure 4 above.

7. Minor points: we have improved the grammar and word usage and also rephrased the "RNA polymerase II transcripts" in the figure legends. The new phrase is: "normalized against the transcripts of RNA polymerase II gene".

Referee #2

As suggested by the editor we have decided to retain the NMD-UPR feedback loop in the manuscript. As suggested by the editor and this reviewer we performed several crucial experiments to better characterize the model, which clearly strengthened and support the NMD-UPR loop:

a. The NMD-UPR feedback loop mechanism predicts that NMD inhibition together with UPR activation **enhances the response of cells carrying a PTC to readthrough treatment, compared to the effect of each treatment alone (NMD inhibition or UPR activation).** To test this prediction we analysed p133 cells, carrying a PTC in the XLF gene. To evaluate the effect of UPR activation and NMD inhibition on XLF function following readthrough, we analysed the ability of the cells to correct the DNA double stand breaks, reflecting the XLF function. As can be seen in new Figure 9,

the combined treatment indeed led to lower levels of DNA double strand breaks, indicating improved restoration of the XLF function following readthrough under combined NMD and UPR treatments and shedding a new light on the functional role of the NMD-UPR feedback loop.

This experiment adds **a functional aspect** of the NMD-UPR feedback loop, in addition to the molecular aspect shown in the previous version of the manuscript (See Results page 19, starting at line 5 from the top of the page). These important results were added also to the abstract (line 4 from the bottom), and throughout the manuscript (Introduction page 6 line 3 from the bottom) and in the Discussion page 21 line 4 from the top and page 25 8 from the bottom).

b. The NMD-UPR feedback loop model was further investigated in the lymphobalstoid cells of the two sisters, in which we have found significant differences in UPR activation in the proteomic analysis. We have found higher transcript levels of known NMD substrates (Figure 6I) in 6538 cells (in which UPR activation was higher), as expected from the model. These results were added to page 17, 7 lines from the bottom.

c. We have shown in the original version of the manuscript that the level of key UPR factors is increased following NMD inhibition (Figure 6A). In order to test whether these factors are bona fide NMD targets, we looked if they harbour known NMD-triggering features (long 3' untranslated region (3'UTR), an intron more than 55 bases downstream of the stop codon (deep intron), and an upstream open reading frame (uORF)). The results indicate that 3/4 analysed genes have known NMD-triggering features (please see page 15, line 3 from the top). These results indicate that NMD directly regulate the transcript level of UPR factors, revealing another mean by which NMD regulates the UPR.

<u>Specific comments</u>

1. Thanks for this comment. We added to the introduction studies showing the importance of readthrough to cancer (please see page 3 line 8 from bottom).

2. As suggested by the reviewer we have added to the Introduction that readthrough response is also strongly dependent on the stop codon and its nucleotide context (please see page 4 line 10 from the top).

3. DTT and TM are both known UPR activators. In order to establish the effect of UPR on NMD we have used DTT, which has a stronger effect. Since the SPQ assay is not well suited for multiple, timed drug additions, especially when some of the treatments (such as DTT) are borderline toxic and have their own potential for interrupting distinct targets in various pathways, we used tunicamycin in this set of experiments. This explanation was added to the text (please see page 32 line 3 from top).

4. We thank the reviewer for pinpointing to an error in referring to figure 3. It should have been 3B and not 3A. This was corrected in the text (page 19 line 8 from bottom).

The reviewer was concerned by the increased level of gH2AX following DTT treatment. This point is correct, but the results show that the level of gH2AX is reduced following UPR activation and readthrough treatment probably showing the strong readthrough effect. Since gH2AX by itself may not represent only DNA double strand breaks (e.g., it is also phosphorylated following DNA single strand breaks) we have performed immunefluorescent analysis of gH2AX colocalized with 53BP1, for quantification of only DNA double strand break repair (please see new Figure S1 and in the text page 10 line 2 from the bottom). As can be seen in this figure, DTT treatment did not result in a significant increase in the level of the colocolized foci, indicating that the increase seen in the Western, may reflect not only DNA double strand breaks.

As suggested by the reviewer we deleted the word “considerably” from the text.

In order to indicate the reproducibility of the results presented in this figure we performed immunofluorescent analysis of gH2AX colocalized with 53BP1, for quantification of the DNA double strand break repair (please see new Figure S1). Importantly, as can be seen in the figure, UPR activation by DTT treatment together with readthrough by G418 resulted in significantly reduced levels of DNA double strand breaks.

5. We have responded to this comment already at the beginning to the response to this reviewer. Please see above.

6. As suggested by the reviewer we have added to the discussion (page 23 line 2 from top) the results of recent studies showing upregulation of transcripts carrying PTCs following readthough treatments by suppressor tRNAs or amlexanox.

7. Figure 4: We thank the reviewer for this comment. CARS should not appear in the legend to Figure 4. We have now deleted it.

As suggested by the reviewer we have now added experiments showing the effect of siRNA directed against hUPF1 in CFP15a, LIPIN1, P133 cells on the level of the transcripts regulated by NMD (please see new Figure S2 and in the text page 12 line 11 from the top).

8. We have corrected the legends of Figures 5 and 6 accordingly.

Referee #3

Figure 1: As suggested by the reviewer we performed enrichment analysis of Gene Ontology categories (Fisher Exact test with Benjamini Hochberg FDR threshold of 0.02). We analysed the list of 440 significantly changing proteins between the two sisters, which was divided into two groups, of the proteins that are higher in 6537 than 6538 and vice-versa. The full list is provided in Table S2. We have also added this point to the text (please see page 8 last line). As can be seen, there are not many dominant categories in this list. We have chosen to pursue our research with the UPR pathway since we hypothesized that UPR activation modulates the NMD through translational attenuation, as extensively studied in the manuscript.

Figures 2 and 3: As requested by the reviewer we have analysed the effect of G418 by itself on the level CFTR and XLF transcripts and found that their level is not changed (Figure S2 and in the text page 11 last line).

DTT and TM are both known UPR activators. In order to establish the effect of UPR on NMD we have used DTT, which has a stronger effect. Since the SPQ assay is not well suited for multiple, timed drug additions, especially when some of the treatments (such as DTT) are borderline toxic and have their own potential for interrupting distinct targets in various pathways, we used tunicamycin in this set of experiments. This explanation was added to the text (please see new Figure S2B and in the text page 12 line 8 from top).

In addition, as requested by the reviewer we have analysed the level of CFTR transcripts following TM treatment and found that their level is increased, supporting our results in Figure 2 (New Figure S2B and in the text page 12 line 8 from top).

The reviewer asked whether the UPR factors shown in Figure 6A are bona fide NMD targets and harbour known NMD-triggering features. For this we analysed whether these factors harbour long 3' untranslated region (3'UTR), an intron more than 55 bases downstream of the stop codon (deep intron), and an upstream open reading frame (uORF). The results indicate that 3 of the 4 analysed genes indeed have known NMD-triggering features (please see page 15, line 3 from the

top). These results indicate that NMD directly regulate the transcript level of UPR factors, revealing another mean by which NMD regulates the UPR.

Minor points

All the requested textual changes were introduced.

In summary, we have addressed all the points raised by the reviewers and added a substantial amount of new data to support our experiments and strengthen the implications of the NMD-UPR feedback loop on readthrough treatments. Indeed the manuscript is now more complete and is significantly improved by the new data. We therefore hope that the revised version will be found suitable for publication in EMM.

Phosphopeptide Table:

Phosphorylated peptides were enriched with titanium dioxide and analysed by LC-MS/MS, with SILAC quantification (as described in the manuscript).Table shows the phosphopeptides that changed more than 1.4-fold between the two cell lines, and are annotated as related to NMD or UPR processes.

Gene name	Amino acid	position	Phosphopeptide	Ratio 6537/6538
ACIN1	S, S	710, 714	_RLS(ph)QPES(ph)AEK_	1.6885
ACIN1	S	240	_LS(ph)EGSQPAEEEEDQETPSR_	2.2094
ACIN1	S	243	_LSEGS(ph)QPAEEEEDQETPSR_	2.2094
ACIN1	S	838	_GVPAGNS(ph)DTEGGQPGRK_	1.5039
RBM8A	S	42	_GFGS(ph)EEGSR_	1.75975
SMG1	T	3578	_NLAT(ph)SADTPPST(ph)VPGTGK_	1.8504
SMG1	S, T	3571, 3574	_NLATS(ph)ADT(ph)PPSTVPGTGK_	1.8507
SMG7	S	735	_S(ph)PPHHSGFQQYQQADASK_	2.0354
SRRM1	S, S	207, 209	_S(ph)RS(ph)PSPAPEK_	0.55136
SRRM1	S	754	_SVS(ph)GS(ph)PEPAAK_	0.59068
SRRM1	S	775	_KPPAPPS(ph)PVQSQS(ph)PSTNWSPAVPVK_	2.25205
SRRM1	S	769	_KPPAPPS(ph)PVQSQSPSTNWSPAVPVK_	2.38575
SRRM1	S	773	_KPPAPPS(ph)PVQS(ph)QSPSTNWS(ph)PAVPVKK_	2.9001
SRRM1	S	260	_VPKPEPIPEPKEPS(ph)PEK_	1.84785
SRRM1	T	416	_TRHSPT(ph)PQQSNR_	2.17925
SRRM1	T	778	_KPPAPPS(ph)PVQS(ph)QSPST(ph)NWSPAVPVKK_	2.5577
SRRM1	S	707	_GAS(ph)SS(ph)PQR_	2.0462
SRRM1	S	452	_RES(ph)PS(ph)PAPKPR_	2.0484
SRRM1	S	389	_LS(ph)PSAS(ph)PPR_	2.25425
HSP90AB1	S	226	_EKEIS(ph)DDEAEEEK_	0.711
HSP90B1	S	306	_EES(ph)DDEAAVEEEEEEK_	0.45219
UBE2J1	S	268	_RLS(ph)TS(ph)PDVIQGHQPR_	1.69835

2nd Editorial Decision 18 February 2014

Thank you for the submission of your manuscript to EMBO Molecular Medicine. We have now heard back from the three Reviewers, whom we asked to re-evaluate your manuscript.

You will see that while Reviewers 1 and 3 are now supportive, Reviewer 2 still not satisfied that all issues raised were adequately addressed.

Reviewer 2 is specifically concerned about a critical point previously raised by Reviewer 3, namely to more convincingly demonstrate that readthrough of the nonsense mutation by G418 is improved when UPR is activated, by showing the level of CFTR or XLF mRNAs and CFTR or XLF proteins by RT-qPCR and Western-blotting. Reviewer 2 notes that the level of readthrough proteins remains to be directly shown (western blotting) and is required as direct evidence.

We have now re-discussed your manuscript in the light of this comment and agree that the Reviewer's point has merit and would thus encourage you to provide the information requested, by carrying out further experimentation if data are not available.

Although it is EMBO Molecular Medicine policy to allow a single round of revision only, I am prepared in this case to allow you to submit a re-revised version as outlined above. I believe that ultimately this would strengthen and consolidate your findings and thus increase the value and impact of your work.

As you know, EMBO Molecular Medicine has a "scooping protection" policy, whereby similar findings that are published by others during review or revision are not a criterion for rejection. However, I do ask you to get in touch with us after three months if you have not completed your revision, to update us on the status. Please also contact us as soon as possible if similar work is published elsewhere.

I look forward to seeing a revised form of your manuscript as soon as possible.

***** Reviewer's comments *****

Referee #1 (Remarks):

The authors have done a commendable job in expanding their study and addressing the points raised in the original round of critiques. The data now provide much better support for the model that is presented and I believe that the study should have broad impact.

Referee #2 (Remarks):

This new submission is clearly improved in comparison to the previous one. The authors answered almost all the major comments made during the first round of review. However, they did not answer one point raised previously by referee 3. They were asked to demonstrate that G418 better stimulates stop codon readthrough efficiency for CFTR or XFL genes upon UPR activation, because the analysis of the function of CFTR or XLF is complementary but not the direct demonstration of the read through activity.
In this revised version they analysed the level of mRNA in presence of G418, but there is no indication of the protein level obtained by stop codon read through. In my opinion this is absolutely essential for the demonstration of a direct effect of the UPR pathway onto stop codon readthrough efficiency. As previously stated by referee 3, By analyzing only the protein function, authors include several additional steps (maturation, transport and localization,...) that can interfere with the

interpretation of the data for the readthrough activity. The authors must perform this critical experiment before the acceptance of their manuscript.

Referee #3 (Comments on Novelty/Model System):

The link between ER and NMD is not new. However the work presented here brings more details and extends the relationship between UPR and NMD. All data are supported by validated statistical tests making high quality manuscript.

Referee #3 (Remarks):

All my questions and concerns received an answer in the revised version. I think the manuscript is stronger and suitable for publication. It is a very nice work and of a great interest for scientists in the field.

2nd Revision - authors' response 20 February 2014

Thank you very much for your mail and the fast response to our revised manuscript.

Few weeks we received an antibody against the CFTR protein which enabled us to perform the requested Western. We have now completed the experiment and added as requested the Western results to the manuscript (new Figure S6 and added the results to the text (page 9 three lines from the bottom).

We also manage to complete the Table summarizing the number (n) of independent experiments underlying each data point, and the actual P value for each test.

As requested, we also have written the section "Paper explained" according to the journal's format.

Within an hour or so we will upload the new files to the EMM website.

We believe that with this last required data the paper will be found suitable for publication in EMM.

Thank you for your assistance along the way,

3rd Editorial Decision 21 February 2014

Thank you for the submission of your revised manuscript to EMBO Molecular Medicine. I am pleased to inform you that I am now ready to accept your manuscript for publication pending the following final minor amendments/requests:

1) We would need a short list (up to 5) of bullet points that summarize the key NEW findings. The bullet points should be designed to be complementary to the abstract and will be used online in our new web platform. Please provide as a separate file and not within the manuscript text.

2) Unfortunately we note that the quality of the images in Fig. 3 and Fig. 5 is poor, specifically the blots are noticeably blocky/blurry when one zooms in. This will cause issues during the production process and should therefore be remedied now with provision of better images.

3) We are now encouraging the publication of source data, particularly for electrophoretic gels and

blots, with the aim of making primary data more accessible and transparent to the reader. Would you be willing to provide a PDF file per figure that contains the original, uncropped and unprocessed scans of all or at least the key gels used in the manuscript? The PDF files should be labeled with the appropriate figure/panel number, and should have molecular weight markers; further annotation may be useful but is not essential. The PDF files will be published online with the article as supplementary "Source Data" files. If you have any questions regarding this just contact me.

Please submit your revised manuscript within two weeks. I look forward to seeing the final version of your manuscript as soon as possible.

© EMBO
